# A Rigid Supramolecular Solution to a Flexible Problem: A Multifunctional Calix[4]arene-Based Strategy to Prevent α‑Synuclein Toxicity

**DOI:** 10.1021/acscentsci.5c02416

**Published:** 2026-05-04

**Authors:** Davide Dell’Accantera, Giulia Piccinini, Cristina Ciabini, Isabella C. Felli, Stefano Volpi, Nelson Marmiroli, Francesco Sansone, Roberta Ruotolo

**Affiliations:** † Department of Chemistry, Life Sciences and Environmental Sustainability, University of Parma, Parco Area delle Scienze 11/A, 43124 Parma, Italy; ‡ Magnetic Resonance Center (CERM) and Department of Chemistry “Ugo Schiff”, 9300 University of Florence, Via L. Sacconi 6, 50019 Sesto Fiorentino, Italy; § Consorzio Interuniversitario Nazionale per le Scienze Ambientali (CINSA), University of Parma, Parco Area delle Scienze 11/A, 43124 Parma, Italy

## Abstract

The pathological aggregation of α-synuclein (syn) is a hallmark of Parkinson’s disease (PD) and related synucleinopathies. In the present study, we report a supramolecular strategy to reduce syn aggregation and its associated cellular toxicity using a panel of rationally designed anionic calix[4]­arenes. Among them, **CLXP1**, a tetraphosphonato derivative with a rigid, preorganized cavity, emerged as a potent inhibitor of syn aggregation in a validated yeast model of PD based on syn overexpression. **CLXP1** significantly improved cell viability by reducing the formation of toxic intracellular inclusions of syn and restoring multiple dysregulated molecular pathways while preserving mitochondrial morphology and redox and lipid homeostasis and enhancing autophagic clearance. NMR analyses revealed that **CLXP1** interacts with key residues of the N-terminal domain of syn, critically involved in membrane anchoring and oligomerization, supporting a model in which this interaction stabilizes the α-helical, membrane-bound conformation of syn, thereby preventing its progression toward toxic oligomeric species. These findings highlight the potential of supramolecular host–guest chemistry to selectively target intrinsically disordered proteins and provide a promising scaffold for the development of new modulators of protein aggregation.

## Graphical abstract

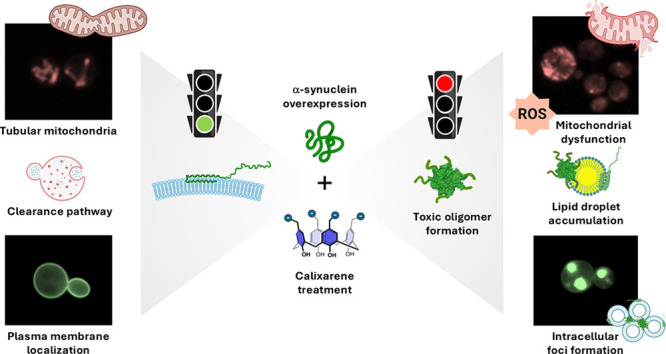

## Introduction

Parkinson’s disease (PD) is a neurodegenerative disorder characterized by the progressive loss of dopaminergic neurons in the substantia nigra pars compacta of the brain and the accumulation of neuronal intracellular inclusions, known as Lewy bodies (LB). These cytosolic inclusions are primarily composed of lipid vesicle clusters, fragmented organelles, and aggregates of α-synuclein (syn), a small protein highly expressed in the human brain, predominantly localized at presynaptic nerve terminals.^1–3^ The involvement of syn in PD pathogenesis is further supported by evidence that autosomal dominant forms of this disease are associated with duplication or triplication of the *SNCA* gene, which encodes syn, as well as several point mutations identified in this gene that promote syn aggregation.^4^ Syn-mediated neurodegeneration also occurs in other neurodegenerative diseases collectively referred to as synucleinopathies, including dementia with LB (DLB), multiple system atrophy (MSA), and the LB variant of Alzheimer’s disease (AD).^5–7^

Although its physiological role is not yet fully elucidated, syn appears to be involved in regulating synaptic plasticity, presynaptic vesicle trafficking, and dopamine release.^2,8^ These functions are closely linked to its structural transitions *in vivo*, as syn is an intrinsically disordered protein with a random coil conformation in solution,^9,10^ which adopts a partially folded structure upon interaction with membranes.^11,12^ In the membrane-bound state, its lysine-rich N-terminal domain (residues 1–60) forms an amphipathic α-helix, stabilized by interactions with lipid bilayers.^11,13–15^ This region contains several imperfect repeats with a KTKEGV consensus motif, which are critical for membrane binding of syn through electrostatic interactions with negatively charged phospholipids.^11,15,16^ These motifs are also known to influence structural flexibility of syn and its propensity to form β-sheet-rich aggregates.^15,17–19^ The central region of syn (residues 61–95), known as the nonamyloid-β-component (NAC) domain, has a highly hydrophobic sequence, initially identified in senile plaques of AD patients.^20^ Under physiological conditions, in cooperation with the N-terminal domain, this region contributes to the formation of the α-helical structure upon lipid binding.^8^ Under pathological conditions, however, its intrinsic propensity to adopt β-sheet-rich conformations drives syn aggregation.^21,22^ In contrast, the C-terminal domain (residues 96–140) remains disordered even upon membrane interaction. This acidic, flexible region appears to act as a molecular chaperone, capable of binding metals, small molecules, and other proteins.^23,24^ Importantly, several studies suggest that the C-terminal domain may exert a protective role against syn aggregation via transient electrostatic interactions with the N-terminal domain or, more likely, through intermolecular electrostatic repulsions.^16,23^

Environmental changes, increased protein expression, and pathogenic mutations can modulate the membrane affinity of syn, thereby promoting its aggregation.^16^ Syn aggregation initially leads to the formation of oligomers, which are widely considered the primary neurotoxic species rather than their amyloid fibrillar counterparts.^25–27^ Syn oligomers can interact with biological membranes, disrupting their integrity and affecting vesicular trafficking.^28–30^ The formation of toxic oligomers with similar structural features and mechanisms of action is also implicated in other neurodegenerative diseases.^31–33^ Notably, several reports suggest that the conversion of these oligomers into more structured amyloid fibrils may represent a cytoprotective mechanism.^27,31,34^

Multiple *in vitro* and animal models have been developed to study synucleinopathies and elucidate the mechanisms of syn-induced proteotoxicity.^35–38^ Among these, *Saccharomyces cerevisiae* has been widely used for over two decades as a robust model system for studying syn aggregation and for high-throughput drug screening, contributing to uncovering the molecular mechanisms underlying the pathology.^37,39,40^ Despite lacking a nervous system, yeast cells overexpressing wild-type syn (HiTox strain) recapitulate several key pathological features of PD, including impaired vesicle trafficking and protein clearance pathways, mitochondrial dysfunction, oxidative stress, and the formation of intracellular foci reminiscent of LB observed in patient neurons.^7,37,41,42^

Several chemical approaches have been explored to counteract syn toxicity, including the use of small-molecule inhibitors,^43^ dendrimers,^44^ peptides,^45^ nucleic acids,^46^ inorganic nanoparticles,^47^ and supramolecular receptors.^48,49^ The latter operate via a host–guest recognition mechanism, targeting noncovalent interactions that drive amyloidogenic protein self-assembly. By selectively including amino acid side chains (or portions thereof) within cavities or pseudocavities, these systems can effectively interfere with peptide/protein aggregation pathways.^50^

In this regard, calix[4]­arenes, a well-studied class of synthetic macrocycles, have proven effective in recognizing lysine and arginine side chains within peptides and model proteins, especially when negatively charged units like sulfonate or phosphonate groups are incorporated at their upper rim.^51–57^ The complexation of lysine side chains on the cytochrome c surface,^53,54,56,57^ for example, involves electrostatic forces and hydrogen bonding between the anionic groups of the calixarene and the ε-ammonium moiety of lysine. Additionally, the lipophilic cavity formed by the aromatic rings of the calixarene is crucially involved in the recognition process, allowing CH−π and cation−π interactions with the methylene groups of the amino acid side chain and inducing a pronounced hydrophobic effect that further stabilizes the lysine–calixarene complex. Reductions in calix[4]­arene cavity rigidity have been reported to cause drastic changes in amino-acid recognition modes,^58,59^ generally leading to significant losses in binding affinity.^60^ Moreover, while X-ray and NMR data reveal lysine and arginine recognition as a general feature of the interactions between anionic calix[4]­arenes and globular proteins, a certain degree of selectivity for some specific residues is often observed, typically due to their higher accessibility on the protein surfaces.^54–57^

Calixarenes have recently also emerged as potential modulators of intrinsically disordered proteins. In one study, Crowley et al.^61^ demonstrated that a tetrasulfonatocalix[4]­arene binds mutant lectins, inducing a ligand-mediated rearrangement of the protein architecture upon recognition of an N-terminal methionine-lysine disordered motif. In another investigation, Gao et al.^62^ reported a coassembled nanosystem including cationic amphiphilic calix[5]­arenes and cyclodextrins that alleviated motor deficits and prevented loss of dopaminergic neurons in 6-hydroxydopamine-treated rats, while also reducing syn fibril formation *in vitro*. In this case, the positively charged calixarenes primarily facilitated cell penetration and the interactions with the negatively charged C-terminal domain of syn, whereas the suppression of syn toxicity was proposed to arise from the action of the cyclodextrin components.

On the basis of this evidence, in the present study we used *S. cerevisiae* cells overexpressing syn as a model of PD^37,39,40,47,63^ to investigate the properties of a selected group of negatively charged calix[4]­arenes (Figure 1), with the aim of identifying promising compounds capable of counteracting syn-induced toxicity.

**Figure 1:**
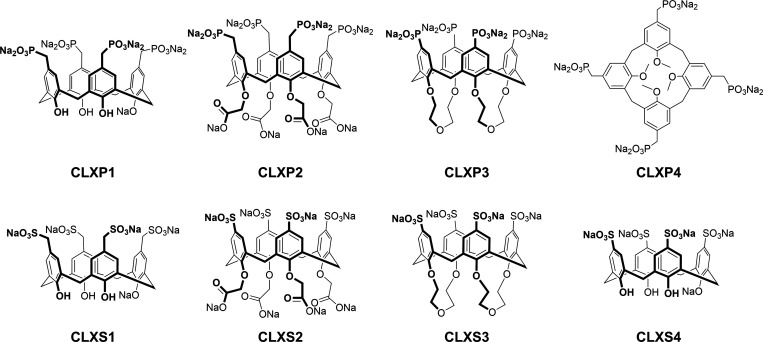
Structures of anionic calix[4]­arenes used in this study.

## Results and Discussion

### Synthesis of Anionic Calix[4]­arenes

Anionic calix[4]­arenes with a preorganized and lipophilic cavity are excellent candidates for the selective complexation of positively charged species like the side chain of the lysine residues,^54,56,57^ which are highly abundant in the N-terminal domain of syn. Given the critical role of these residues in the pathological aggregation of syn,^15^ we selected a series of calix[4]­arenes functionalized at the upper rim with phosphonate or sulfonate units (Figure 1) to identify new modulators of syn-induced toxicity. The lower rims of these derivatives were functionalized using different synthetic approaches that are known to ensure the formation of a discrete and sufficiently rigid macrocyclic cavity. The only exception is represented by **CLXP4** (Figure 1), which features high conformational mobility due to the presence of methyl substituents on its phenolic oxygen atoms. These substituents, in fact, preclude the formation of hydrogen bonds at the lower rim and are sufficiently small to allow free rotation of the aromatic rings within the macrocyclic annulus, making this calixarene a control compound for evaluating the influence of scaffold rigidification on the interaction with syn.

**CLXP1**, **CLXS1**, and **CLXS4** (Figure 1) exhibit a highly preorganized cavity due to the hydrogen-bonding network formed by hydroxyl groups at the lower rim. At physiological pH, one of the four hydroxyl groups is deprotonated, further reinforcing the array and the deriving structure.^64^ This network prevents conformational exchange between flattened cone isomers, ensuring consistent cavity accessibility and efficient guest complexation. Calix[4]­arenes **CLXP2** and **CLXS2** (Figure 1) are instead functionalized at the lower rim with four methylcarboxylate units, and a significant structural rigidity is achieved through the complexation of sodium ions by the phenolic oxygens and carboxylate units.^57^ Experimental evidence indicates that this metal ion complexation enhances the structural integrity of the cavity, facilitating accommodation of the lysine side chain. Notably, previous studies demonstrated that, under these conditions, **CLXS2** selectively binds lysine residues within the structure of cytochrome c.^57^ Another approach to preorganize the calix[4]­arene cavity based on the lower-rim functionalization with short bridges connecting vicinal phenols (positions 1,2 and 3,4) was used for compounds **CLXP3** and **CLXS3** (Figure 1). Our previous studies,^65^ in fact, showed that a bis-crown-ether calix[4]­arene can include the aromatic moiety of α-amino acids and ammonium salts due to its highly restricted conformational mobility, surpassing a tetrapropoxy analogue that undergoes conformational exchange between two cone conformers with *C*2*v* symmetry and therefore lacks an effective cavity to host the substrate.

**CLXS1** and **CLXS4** were synthesized following previously reported protocols.^66,67^ **CLXP1** was prepared starting from the chloromethylated derivative **1** ^68^ (Scheme 1). The first step involved the preliminary substitution of the chlorine atoms with iodine using sodium iodide in acetone (Finkelstein reaction), followed by an Arbuzov reaction with triethyl phosphite at room temperature (Scheme 1). Notably, this modified method reduced the required amount of triethyl phosphite compared with conventional protocols,^68^ facilitating the isolation of the tetrakis­(diethyl)­phosphonate derivative **2** (Scheme 1).^69^ The final deprotection step was carried out using 12 M hydrochloric acid (HCl) in 1,4-dioxane. The resulting compound was treated with sodium hydroxide in water to give corresponding anionic derivative **CLXP1**.

**Scheme 1:**
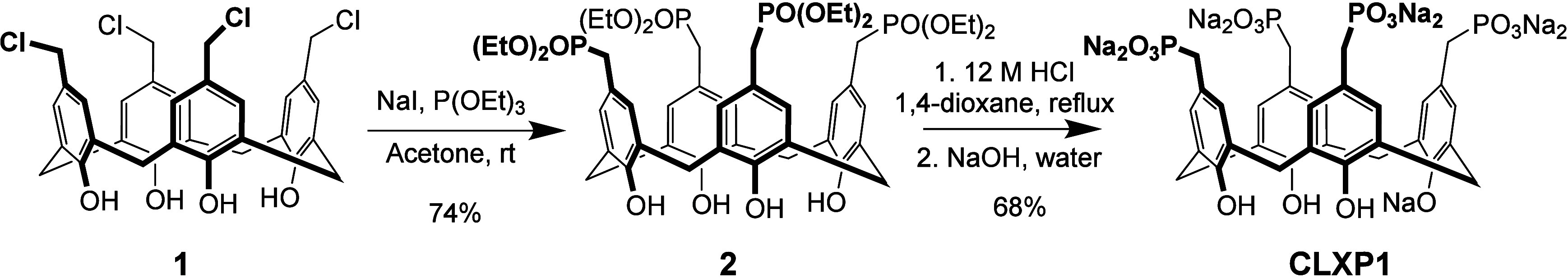
Synthesis of **CLXP1**

**CLXP2** and **CLXS2**, both characterized by the presence of carboxylate groups at the lower rim, were synthesized by using two different approaches. The synthesis of **CLXP2** started from tetraformylcalix[4]­arene **3** (Scheme 2), performing lower rim alkylation using ethyl bromoacetate and sodium carbonate in acetonitrile to give derivative **4** in cone conformation. Subsequent reduction of the formyl derivative **4** to the corresponding alcohol derivative **5** was performed using sodium borohydride. The chlorination of compound **5** to obtain compound **6** was carried out using thionyl chloride, followed by a combined Finkelstein–Arbuzov protocol (vide supra) to give **7**. Hydrolysis of the phosphonate and carboxylate esters was achieved by treatment with 12 M HCl in dioxane, followed by neutralization with sodium hydroxide, resulting in the formation of compound **CLXP2**.

**Scheme 2:**
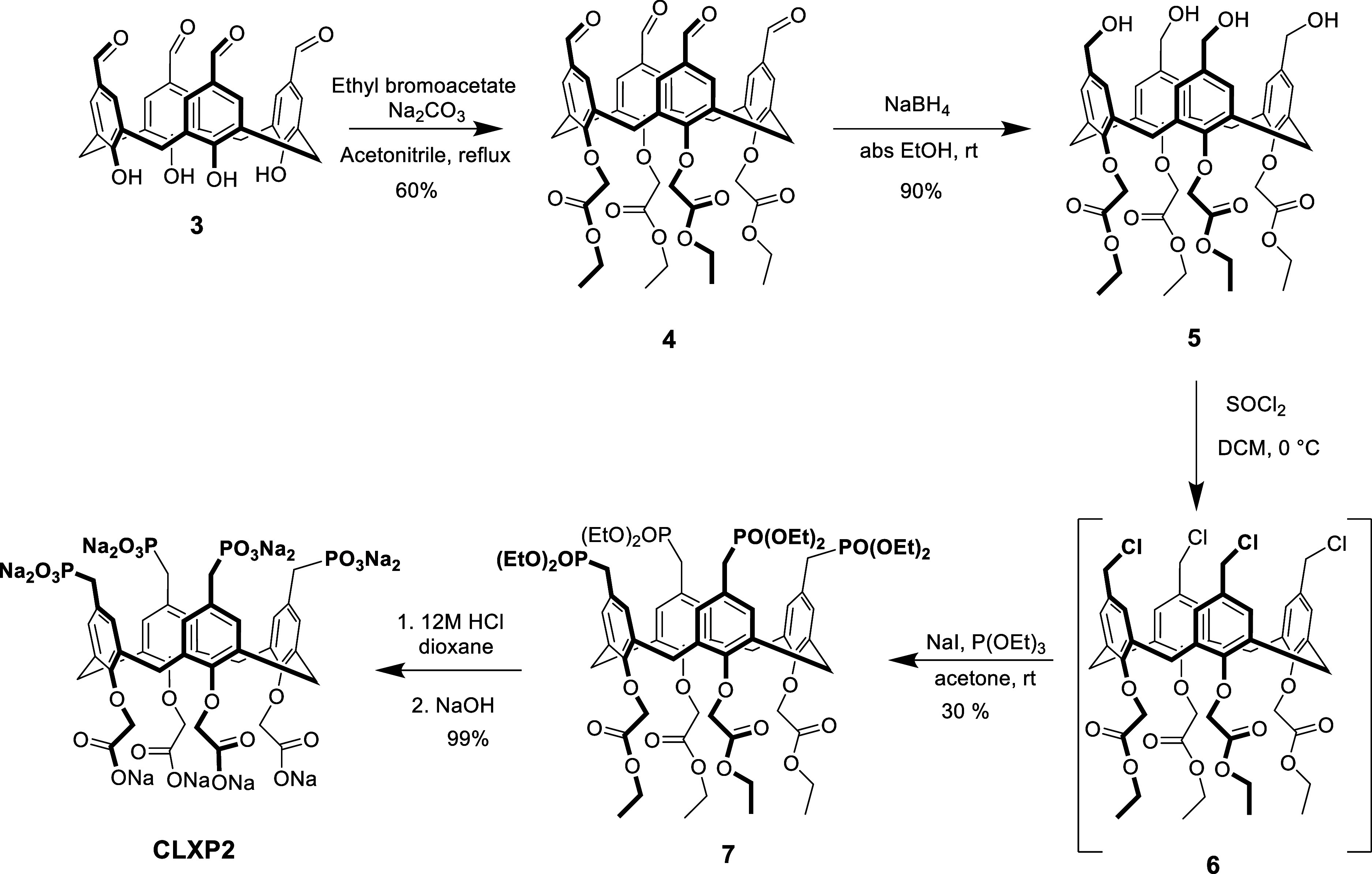
Synthesis of **CLXP2**

**CLXS2** was synthesized following the literature procedures.^70^

For **CLXP3** and **CLXS3**, the rigid cone-shaped structure of the calixarene is provided by two short di­(ethylene glycol) chains, each of them tethered to the oxygen atoms of two adjacent phenolic rings, also referred to as positions 1,2 and 3,4. **CLXP3** was synthesized in its fully protonated form following an already published procedure^52^ and then transformed into the corresponding salt. **CLXS3** was synthesized using the method described by Daze et al.;^60^ despite this, in our hands, the final product was directly obtained upon treating its crude chlorosulfonated precursor with ice rather than performing separated chlorosulfonation and hydrolysis steps. As for the other macrocycles, treatment with NaOH produced the desired final product **CLXS3**.

The synthesis of **CLXP4** (Scheme 3) started from compound **8**, prepared according to the method reported by Shinkai et al.^71^ An Arbuzov reaction was conducted on **8** using neat tris­(trimethylsilyl)­phosphite at 100 °C in a Schlenk flask, followed by methanolysis to remove the trimethylsilyl groups from the phosphonate units and treatment with sodium hydroxide to yield **CLXP4**. The ^1^H NMR spectrum in D_2_O of **CLXP4** [see the Supporting Information (SI)] clearly showed the presence of several species corresponding to different possible geometries of the calix[4]­arene derivative in slow exchange on the NMR time scale. The major conformer is the so-called 1,3-alternate, where each aromatic ring adopts opposite orientation with respect to the adjacent ones. The singlet generated by the CH_2_ methylene bridges at 3.70 ppm is diagnostic for this conformation and the signal at 35.9 ppm in the ^13^C NMR spectrum for the corresponding carbon atoms further supports this conclusion.^72^ Relevant for our present study is the demonstration that **CLXP4** predominantly adopts in aqueous solution a conformational arrangement which lacks a defined macrocyclic cavity.

**Scheme 3:**
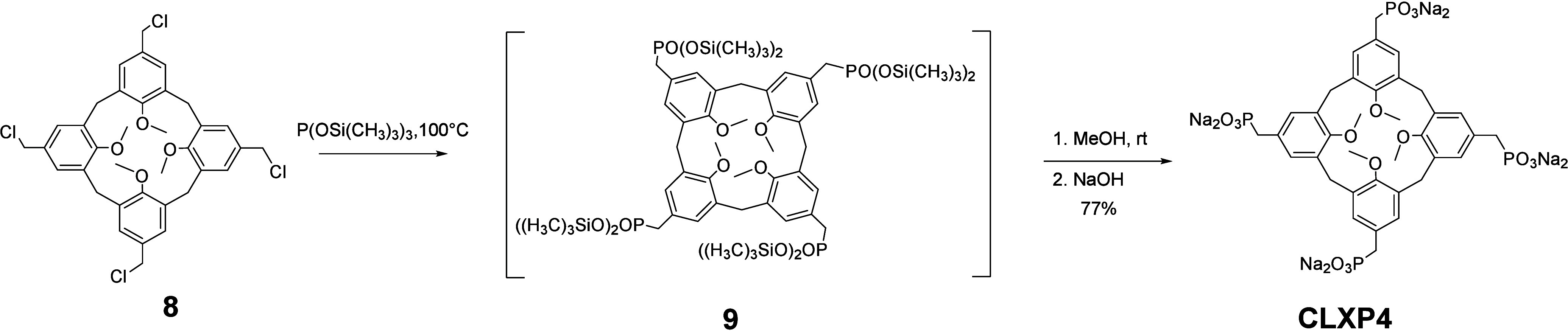
Synthesis of **CLXP4**

### **CLXP1** Protects Yeast Cells from Syn-Induced Toxicity

To identify calix[4]­arene-based compounds (Figure 1) capable of mitigating the toxic effects of syn, we utilized a validated yeast model of PD (HiTox strain) overexpressing human syn fused with green fluorescent protein (GFP) under the control of the *GAL1* promoter.^37,39,47^ Previous studies have shown that GFP tagging does not alter the aggregation properties of syn and that the fusion protein displays localization patterns in yeast comparable to those of the untagged protein.^7,37,73^ In galactose-containing medium (inducing condition; SGal medium), the HiTox strain strongly overexpresses syn, leading to the formation of cytoplasmic inclusions (Figure S1A) consisting of syn aggregates^73^ associated with clusters of lipid vesicles, which block vesicular trafficking and lead to cell death, mimicking the cellular pathology observed in PD.^3,7,37,41,42^

The formation of cytoplasmic inclusions occurs when the syn monomer concentration exceeds a critical threshold for aggregation^7,42,74^ through a two-step process involving an initial interaction with the plasma membrane mediated by the N-terminal domain of syn, followed by the formation of intracellular foci driven by the NAC domain.^7,73^ In line with these observations, syn protein mutations that impair plasma membrane binding and intracellular foci formation are associated with reduced toxicity in yeast.^7,75,76^

After a few hours of incubation under inducing conditions, small intracellular inclusions become detectable near the plasma membrane in the HiTox strain (Figure S1A). These small aggregates increase in size over time and can coalesce into larger foci (Figure S1A), a process that correlates with a rapid loss of proliferative capacity in yeast, indicative of syn-induced toxicity.^7,42,63^

Accordingly, we found that, after 48 h of growth in SGal medium, the majority of syn-overexpressing cells (∼80%) were GFP-negative (GFP^–^) but positive for propidium iodide (PI) staining (PI^+^), indicating loss of membrane integrity and necrosis-like cell death induced by prolonged protein overexpression (Figures 2 and S1B).^77^ Moreover, more than 90% of the few remaining viable cells (GFP^+^) exhibited large intracellular syn foci (Figure 2A–C) and an altered morphology characterized by cellular swelling, loss of the typical ellipsoidal cell shape, and aberrant budding (Figure S1C). All these features are reminiscent of senescence-associated phenotypes previously described in yeast.^78^

**Figure 2:**
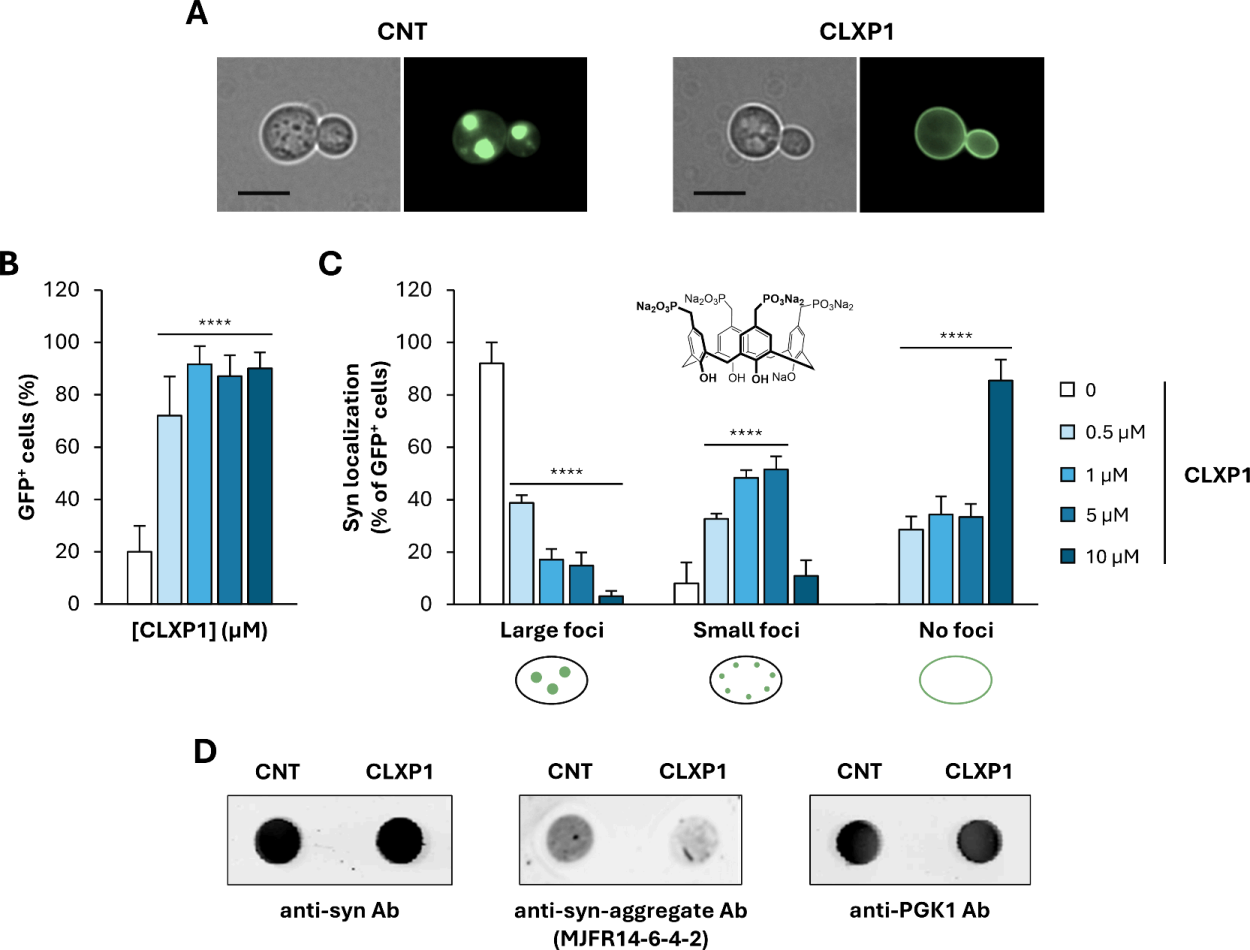
**CLXP1** counteracts syn-induced toxicity by reducing intracellular foci formation in the HiTox strain. (A) Microscopy analysis of cells overexpressing syn-GFP (HiTox strain) grown for 48 h at 28 °C in the presence or absence of **CLXP1** (10 μM). For each condition, both phase-contrast (*left*) and fluorescence (*right*) images are shown. Scale bars: 5 μm. (B) Quantification of GFP-positive (GFP^+^) cells after 48 h of treatment with increasing concentrations of **CLXP1** (0.5–10 μM). Data are expressed as percentages relative to the total number of cells. (C) Percentage of GFP^+^ cells displaying intracellular foci (large or small) or no detectable foci (with syn predominantly localized at the plasma membrane) calculated relative to the total number of GFP^+^ cells. (D) Dot blot analysis of whole-cell extracts (50 μg) from untreated and **CLXP1**-treated cells, performed using anti-syn and anti-syn aggregate (MJFR14-6-4-2) antibodies (Ab). Immunoreactivity of the constitutively expressed phosphoglycerate kinase (PGK1) enzyme was used as a loading control. CNT indicates the control (untreated) sample. Statistical significance was determined using one-way ANOVA followed by Bonferroni’s multiple comparisons test (****, *p* < 0.0001).

Yeast-based chemical screenings revealed that some of our calix[4]­arenes functionalized with phosphonate groups significantly reduced both the formation of intracellular foci and syn toxicity (Figures 2 and S2). Among these, **CLXP1** emerged as the most effective compound, markedly enhancing the proportion of viable fluorescent cells (GFP^+^),^63^ even at submicromolar concentrations, and inducing a dose-dependent reduction of intracellular syn foci (Figure 2A–C). At the most effective dose (10 μM), the majority of GFP^+^ cells (∼90%) did not show intracellular foci and instead displayed syn predominantly localized at the plasma membrane (Figure 2A–C), a phenotype associated with suppression of syn toxicity in both genetic and chemical screens.^41,63,79,80^ Notably, **CLXP1**-treated cells also displayed diffuse cytosolic fluorescence in addition to plasma membrane localization (Figure 2A), suggesting that under these conditions syn may exist in a dynamic equilibrium between a nonaggregated, intrinsically disordered state and a membrane-bound conformation, as observed in healthy neuronal cells.^37,81^ In addition, at the most effective dose, the majority of **CLXP1**-treated cells retained the typical ellipsoidal morphology of healthy, proliferating yeast cells, in contrast to untreated cells (Figure S1C).

To assess proliferative capacity following syn expression, yeast cells grown under inducing conditions (SGal medium) for 48 h at 28 °C were harvested, diluted, and plated onto noninducing conditions (glucose-containing agar plates; SD medium). The clonogenic assay revealed a ∼10-fold increase in colony-forming units (CFU) in **CLXP1**-treated samples compared to untreated controls, indicating a higher proportion of metabolically active cells capable of forming colonies after **CLXP1** exposure.

Importantly, immunoblot analysis showed that the protective effect of **CLXP1** was not due to reduced syn expression levels (Figure 2D). To further assess whether **CLXP1** modulates the aggregation state of syn, whole-cell extracts obtained from untreated and **CLXP1**-treated cells at the most efficient dose (10 μM) were analyzed by dot blot using the conformation-specific antibody MJFR14-6-4-2, which selectively detects syn oligomers.^82^ Immunological analyses revealed a marked (∼5-fold) reduction in toxic oligomeric syn species following **CLXP1** treatment compared to the untreated control (Figure 2D).

Less pronounced protective effects were observed for two of the other phosphonatocalix[4]­arenes, with **CLXP2** and **CLXP3** increasing GFP^+^ viable cells by approximately three and 2-fold, respectively, at their optimal concentration (10 μM), while reducing the formation of large syn foci by ∼4-fold (Figure S2). Unlike **CLXP1**, whose conformational rigidity is conferred by the hydrogen-bonding network involving the phenol hydroxyl groups at the lower rim, **CLXP2** and **CLXP3** are functionalized with methylcarboxylate units or short bridges connecting vicinal phenols (Figure 1). These structural differences may underlie the greater bioactivity of **CLXP1** compared to **CLXP2** and **CLXP3**, possibly reflecting differences in the binding mechanism to syn, a different propensity to accommodate lysine side chain, and the role of free hydroxyl groups in participating in redox reactions *in vivo*. Notably, **CLXP4**, which lacks free hydroxyl groups, structural rigidity and a well-defined macrocyclic cavity for lysine side chain complexation (Figure 1), did not induce significant phenotypic changes compared to untreated cells (Figure S3).

Regarding sulfonatocalix[4]­arenes (Figure 1), **CLXS1** and **CLXS4** bearing free hydroxyl groups at the lower rim displayed cytotoxicity at the highest tested concentration (10 μM), as evidenced by a significant reduction in GFP^+^ cells compared to untreated controls (Figure S4). This is in line with a previous study that showed that sulfonatocalix[4]­arenes possess antimicrobial activity against several fungal strains.^83^ However, at a lower dose (1 μM), both compounds appeared to slightly improve cell viability (∼2-fold increase in GFP^+^ cells), even if large intracellular aggregates were still detected in ∼30% and ∼50% of GFP^+^ cells, respectively (Figure S4).

In contrast, **CLXS2** and **CLXS3**, which present at the lower rim carboxylate groups or short bridges (Figure 1), respectively, did not show significant phenotypic changes compared to untreated cells (Figure S3).

Although the toxicity observed for **CLXS1** and **CLXS4** at high doses highlights the possibility of additional targets for them in yeast, overall, the data seem to support the relevance of free hydroxyl groups at the lower rim for the biological activity of the anionic calix[4]­arenes used in these studies.

### **CLXP1** Reduces Oxidative Stress and Mitochondrial Dysfunction Induced by Syn Overexpression

Hydroxy-calix­[4]­arene derivatives have been reported to exhibit radical- and reactive oxygen species (ROS)-scavenging activity.^84,85^ Given that syn overexpression significantly increases intracellular ROS levels,^42,79,86^ we investigated whether **CLXP1** treatment might reduce ROS accumulation in the HiTox strain. To address this, intracellular ROS levels were quantified using CellROX Orange, a cell-permeant fluorogenic dye that detects a broad spectrum of ROS in living cells. After 4 h of incubation under inducing conditions, treatment with **CLXP1** at its maximal effective dose resulted in a significant reduction (∼60%) in ROS levels compared to untreated controls (Figure 3A).

**Figure 3:**
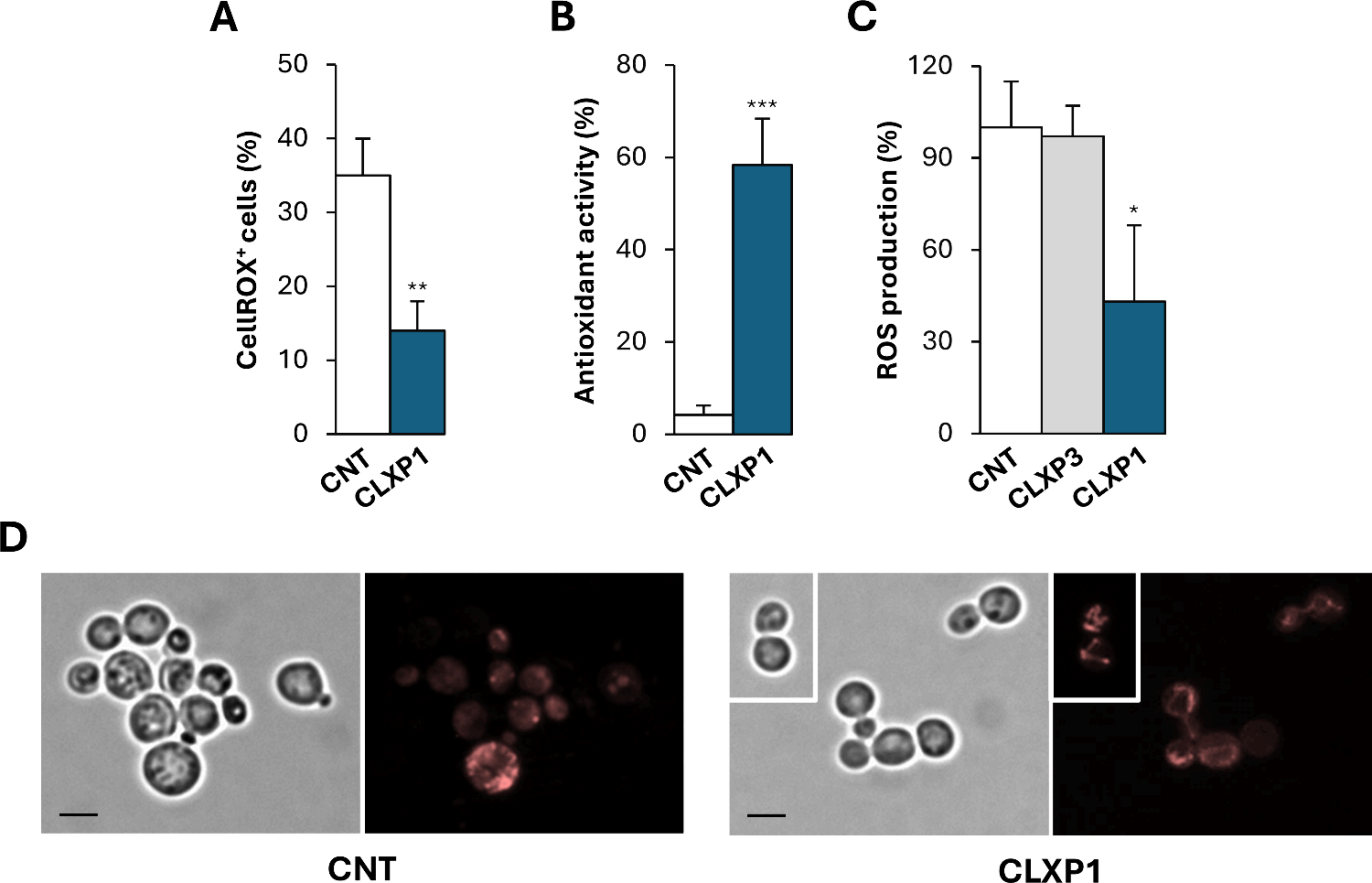
**CLXP1** enhances ROS scavenging activity and mitigates mitochondrial dysfunction in syn-overexpressing cells. (A) HiTox strain was treated with **CLXP1** for 4 h in SGal medium and stained with CellROX Orange Reagent. Fluorescence microscopy was used to visualize CellROX-positive (CellROX^+^) cells, which were then quantified. Statistical significance was assessed using a two-tailed unpaired *t* test (**, *p* < 0.01). (B) Antioxidant activity was measured in whole-cell extracts from untreated or **CLXP1**-treated cells after 24 h in SGal medium using the ABTS assay. Statistical significance was determined using a two-tailed unpaired *t* test (***, *p* < 0.001). (C) ROS production was assessed using the DCFDA assay. Yeast cells grown in SD medium were treated with **CLXP1** or **CLXP3** for 4 h, followed by exposure to *tert*-butyl hydroperoxide (tBHP). ROS levels were then measured. Statistical analysis was performed using one-way ANOVA, followed by Bonferroni’s multiple comparisons test (*, *p* < 0.05). (D) Yeast cells expressing mitochondrially targeted RFP were grown in SGal medium for 24 h with (*right*) or without (*left*) **CLXP1** treatment. For each condition, phase-contrast (*left-side*) and fluorescence (*right-side*) images are shown. CNT indicates the control (untreated) sample. Scale bars: 5 μm.

To investigate whether **CLXP1** treatment enhanced endogenous redox-buffering capacity, we performed the ABTS radical scavenging assay on whole-cell extracts obtained from cells treated or untreated with **CLXP1**. After 24 h of treatment, antioxidant capacity was significantly increased in **CLXP1**-treated samples relative to controls (Figure 3B). To determine whether **CLXP1** exhibited antioxidant activity, independently of its effects on syn aggregation, we evaluated its redox-scavenging capacity under exogenous oxidative stress. Cells were grown in noninducing conditions (SD medium) to repress syn expression, and oxidative stress was induced by treatment with *tert*-butyl hydroperoxide (tBHP). ROS levels were measured using the 2′,7′-dichlorofluorescin diacetate (DCFDA) assay, based on a cell-permeable fluorogenic probe that detects a broad range of oxidative species. **CLXP1** treatment significantly reduced ROS accumulation in this context (Figure 3C), suggesting that this compound combines intrinsic antioxidant activity with enhancement of endogenous redox defenses in the HiTox strain (Figure 3B). In contrast, **CLXP3**, lacking hydroxyl groups at the lower rim, did not confer a comparable protective effect, underscoring the potential role of **CLXP1** functional groups in redox modulation (Figure 3C). These results suggest that **CLXP1**, similarly to other compounds such as polyphenol derivatives,^87^ may combine both antiaggregative and intrinsic antioxidant activities to cooperatively mitigate syn-aggregation-induced oxidative stress.

Previous studies have shown that syn aggregation induces mitochondrial dysfunction, which results in the generation of elevated intracellular ROS levels.^42,79,86^ Oxidative stress can, in turn, promote syn misfolding,^88,89^ creating a pathological feedback loop that further promotes syn aggregation.^90^ To assess mitochondrial dysfunction, we used a HiTox strain expressing a mitochondrial-targeted red fluorescent protein (mtRFP).^47^ In wild-type cells, mitochondria form an interconnected tubular network maintained by balanced fission and fusion dynamics.^47^ After 24 h of induction, syn-overexpressing cells displayed punctate structures, indicative of mitochondrial fragmentation (Figure 3D). Conversely, **CLXP1** treatment restored the tubular mitochondrial architecture, suggesting a rescue of mitochondrial dynamics and morphology (Figure 3D).

As previously observed, untreated cells exhibited marked morphological abnormalities (Figure 3D), hallmarks of senescence-associated phenotypes in yeast,^78^ in contrast to **CLXP1**-treated cells. Consistent with these observations, after 48 h of induction, the untreated HiTox strain markedly acidified the culture medium (pH 5), whereas **CLXP1**-treated cells maintained an extracellular pH comparable to that of the standard yeast medium (pH 6). Aging yeast cells have been reported to acidify their environment through the release of organic acids,^91^ a process that also promotes intracellular acidification.^92^ Acidic intracellular conditions, in turn, have been shown to accelerate specific steps of the syn aggregation process.^93^

Collectively, these data suggest that **CLXP1** exerts cytoprotective effects by restoring redox homeostasis, preserving mitochondrial function, and delaying senescence-associated phenotypes.

### **CLXP1** Modulates Lipid Homeostasis and Clearance Pathways in Yeast

Transcriptomic analyses in the HiTox strain revealed that **CLXP1** markedly upregulates *HSP12* (Figure S5A), a gene encoding a small heat shock protein (sHSP) that interacts with vesicles and lipid droplets (LDs)^94,95^ and maintains membrane stability under stress conditions.^96^ Similar to syn, Hsp12 is an intrinsically disordered protein in solution but adopts an amphipathic α-helical conformation upon binding to negatively charged phospholipids of the plasma membrane or LDs.^96,97^ Recent evidence indicates that human sHSPs, such as Hspb6, reduce syn aggregation by preventing the formation of transient syn–lipid complexes and delaying primary nucleation.^98^ A similar protective mechanism can be envisaged for Hsp12 in yeast. Accordingly, **CLXP1**-mediated overexpression of *HSP12* could contribute to reducing syn-dependent toxicity by modulating syn–membrane interactions and preserving lipid homeostasis.

Dysregulation of lipid metabolism is strongly implicated in the pathogenesis of PD.^99,100^ Syn overexpression has been reported not only to promote lipid vesicle clustering, as described above, but also to induce the accumulation of LDs,^37,101^ which can be readily detected with Nile red staining. Syn interacts with the phospholipid monolayer of LDs, and these lipid-rich microenvironments can in turn promote syn aggregation into oligomeric and fibrillar structures.^30,93,101^ Since Hsp12 binds LDs with high affinity, increased cellular abundance of this protein induced by **CLXP1** exposure could counteract this detrimental feedback loop of syn–LD interactions that promotes syn aggregation.

We found that **CLXP1** treatment significantly reduces Nile red fluorescence after 4 h of growth under inducing conditions (Figure S5B), indicating that the phosphonatocalix[4]­arene improves lipid homeostasis in the HiTox strain by limiting excessive accumulation of LDs. LD degradation via autophagy is known to contribute to lipid homeostasis and support cell viability under conditions of metabolic energy depletion.^102^ Because these clearance pathways can also promote the disposal of toxic oligomeric species and counteract neurotoxicity,^103,104^ we next examined whether **CLXP1** could affect autophagy. Transcriptomic analysis revealed that **CLXP1** treatment upregulates the expression of *ATG17* (Figure S5A), a gene encoding a core autophagy regulator implicated in both early and late stages of the process^105^ as well as in LD degradation.^102^ In contrast, **CLXP1** did not affect the expression of *RPN4*, encoding a master regulator of the ubiquitin-proteasome system (UPS),^106^ suggesting that the calixarene derivative preferentially targets autophagy rather than proteasomal clearance.

To assess the impact of **CLXP1** on syn aggregate clearance, we monitored the reduction of cytoplasmic syn foci in the HiTox strain over time after *GAL1* promoter shut-off.^107,108^ Upon shifting cells from inducing (galactose-containing medium) to repressive (glucose-containing medium) conditions, **CLXP1**-treated cells exhibited markedly accelerated clearance of syn foci compared to untreated controls (Figure S5C), consistent with enhanced autophagic clearance induced by the calix[4]­arene.

### **CLXP1** Interacts with the N-Terminal Domain of Syn

Although previously reported,^56^ we first performed ^1^H NMR titrations in D_2_O to verify the ability of **CLXP1** to bind lysine through inclusion of the amino acid side chain in the calixarene cavity, using a simplified model based on the *N*-acetyl-l-LysGlyOMe dipeptide (see the SI for details). As expected, a significant upfield shift of the CH_2_N protons of Lys upon addition of increasing amounts of calix[4]­arene confirmed the amino acid complexation.^56^ An association constant on the order of 10^3^ M^–1^ was determined and independently validated by ITC titrations (Figures S6–S8).

Subsequently, the interaction between ^15^N-labeled syn and **CLXP1** was investigated by 2D ^1^H–^15^N HSQC NMR (Figures 4A and S9). Upon ligand addition, chemical shift perturbation (CSP) values increased in a concentration-dependent manner (Figure S10A), and the corresponding *K*_d_ values for most well-resolved peaks, assuming a 1:1 interaction model, were determined (Figure S10B). HSQC spectra clearly indicate preferential binding of **CLXP1** to the lysine-rich N-terminal domain of syn (Figure 4B). Evaluation of ^15^N relaxation rates supported the interaction of **CLXP1** with this region: in the absence of ligand, typical values for an intrinsically disordered protein were observed, whereas the addition of **CLXP1** markedly increased ^15^N *R*_2_ values in the N-terminal domain (Figure 4C), likely due to chemical/conformational exchange contributions.

**Figure 4:**
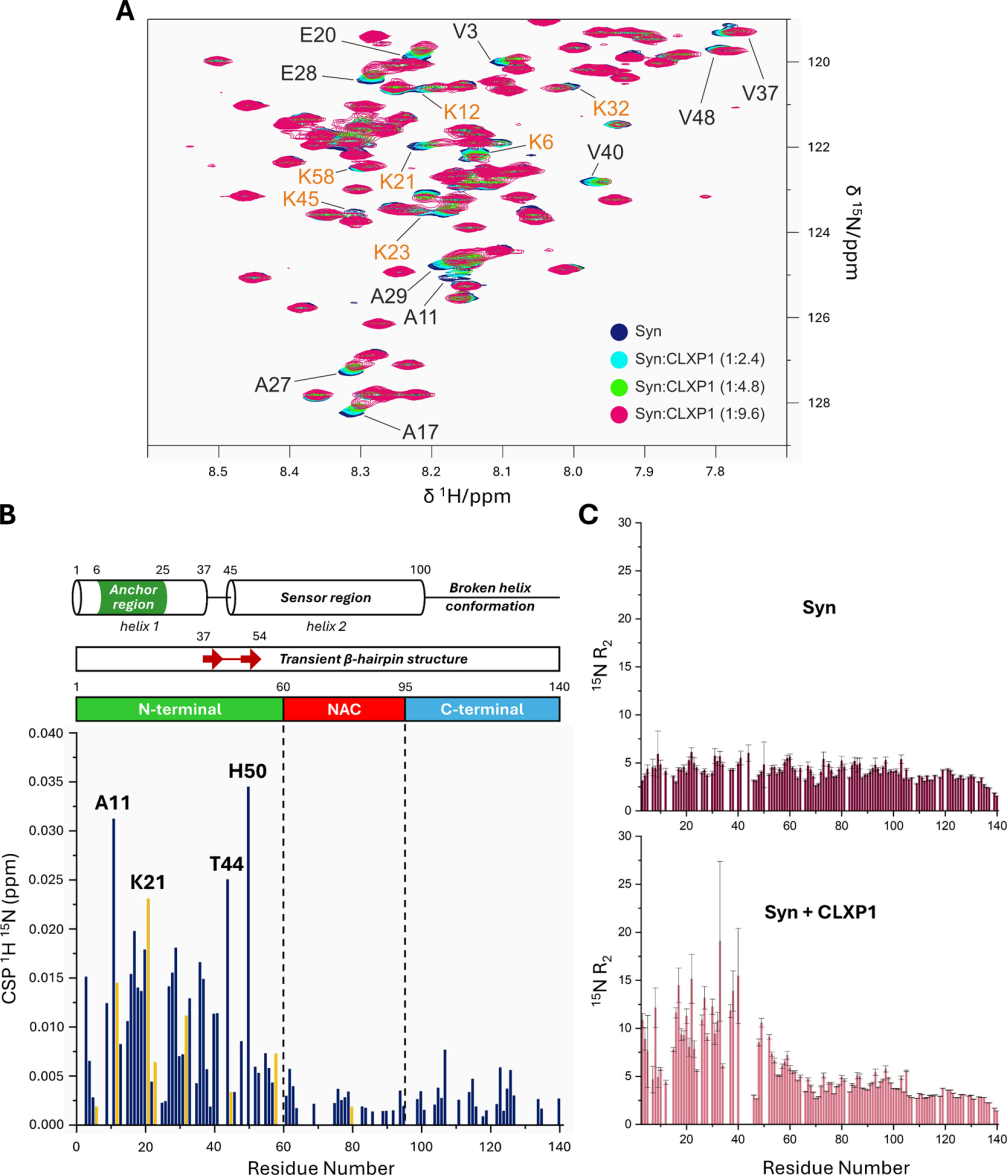
NMR analysis of **CLXP1** binding to syn. (A) Zoom of the most perturbed spectral region of ^1^H–^15^N HSQC spectra of syn (190 μM; 25 mM HEPES, 25 mM KCl, pH 7.2). The figure shows an overlay of spectra acquired in the absence (dark blue) and in the presence of **CLXP1** at syn:**CLXP1** molar ratios of 1:2.4 (light blue), 1:4.8 (green), and 1:9.6 (magenta). The residues most affected by **CLXP1** addition are labeled (lysine residues are marked in orange). (B) Plot of ^1^H–^15^N chemical shift perturbation (CSP) values versus residue number at 1:9.6 syn:**CLXP1** molar ratio. The CSP value reported for residue 41 (Gly) was measured at 2.4 equiv of **CLXP1**, as the corresponding signal broadened beyond detection at higher calixarene concentrations. Orange bars indicate lysine residues affected by **CLXP1** binding. The most perturbed residues are labeled. A schematic representation of syn domain organization is shown at the top of the figure. It includes the N-terminal domain with the membrane “anchor region” (marked in dark green) and the “sensor region”, followed by the central NAC region (marked in red), and the C-terminal region (marked in light blue). The broken α-helix conformation and the transient β-hairpin structure are shown above the syn domain map. (C) Plots of transverse relaxation rates (^15^N *R*_2_) of syn versus residue number in the absence (dark pink) and presence (light pink) of **CLXP1** at a 1:9.6 α-syn:**CLXP1** molar ratio.

As expected, the analysis of the spectra shows that several lysine resonances undergo significant chemical shift changes (Figure 4A,B). The anionic upper rim of **CLXP1** drives the ligand to the positively charged N-terminal region, reasonably supported by the synergistic inclusion of Lys side chain within the macrocyclic cavity through CH−π and hydrophobic interactions. Interestingly, only a subset of lysines was affected, with Lys21 showing the strongest perturbation, consistent with a preferential interaction of **CLXP1** with the N-terminal region of syn.

As previously observed by NMR for anionic calixarenes interacting with proteins such as cytochrome c,^56^ the perturbation induced by **CLXP1** in syn is not restricted at the lysine residues (Figure 4A,B). Rather, it extends to neighboring amino acids and to residues likely affected by ligand-induced conformational rearrangement.

Importantly, some of the most perturbed residues of syn (Ala11 and Lys21; Figure 4B) are located within the region encompassing the first and second KTKEGV consensus motifs (residues 6–25), which has been reported to function as a membrane “anchor” for syn.^81^ This region adopts an α-helical structure with limited mobility and mediates the initial association of syn with lipid membranes (Figure 5).^8,81,109^ It has been proposed that membrane anchoring mediated by this region increases the local concentration of syn at the membrane surface, thereby facilitating pathogenic intermolecular interactions (Figure 5),^8,81,109^ and promoting the formation of toxic oligomers that can act as seeds for further aggregation.^7,8,17,42,75^

**Figure 5:**
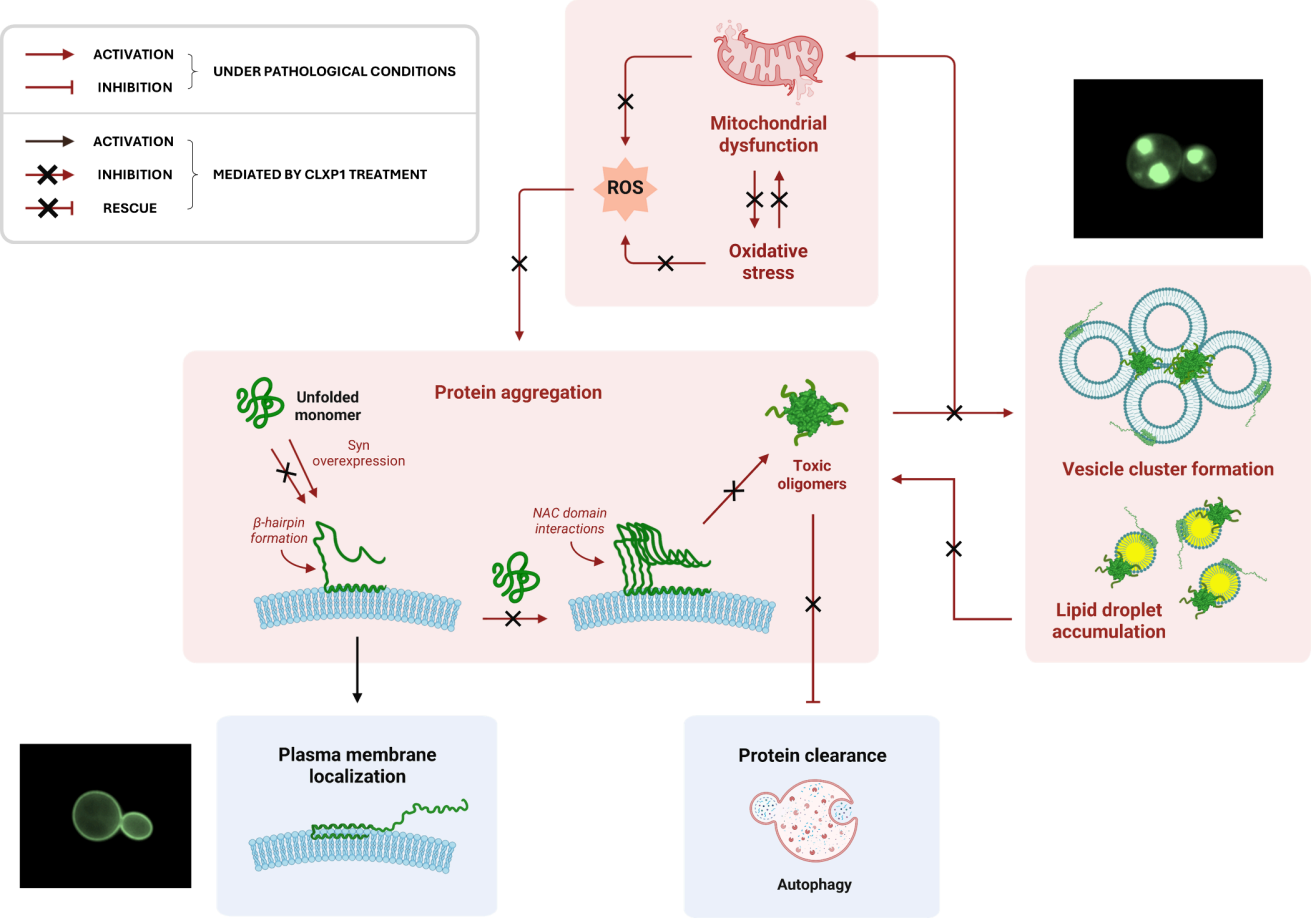
Schematic representation of the proposed mechanism by which **CLXP1** modulates the syn aggregation pathway. Under pathological conditions, syn overexpression promotes protein aggregation, leading to the formation of toxic oligomers that interfere with vesicle trafficking, induce lipid droplet and ROS accumulation, and cause mitochondrial dysfunction and defects in clearance pathways. **CLXP1** treatment counteracts these effects by reducing oligomer formation, restoring multiple dysregulated pathways, and promoting syn retention at the plasma membrane. Fluorescence microscopy images shown in Figure 2A are associated with the corresponding molecular pathways depicted in this schematic representation. Red symbols indicate pathways activated (arrows) or inhibited (T-bars) under pathological conditions (syn overexpression); black arrows indicate pathways activated by **CLXP1** treatment; black crosses denote pathological pathways inhibited by **CLXP1** treatment. The scheme was created with BioRender (biorender.com).

The N-terminal “anchor” region of syn plays a pivotal role in mediating membrane interactions not only of the protein in its monomeric state, but also of oligomeric species and fibrillar aggregates.^74,110^ This interaction facilitates the insertion of β-sheet-rich syn assemblies into the hydrophobic core of lipid layers.^8,74,109^

By targeting this region, **CLXP1** may interfere with syn–membrane association, thereby maintaining a fraction of syn in a cytosolic, intrinsically disordered state and lowering its local concentration at biological membranes, consequently limiting the pathological intermolecular interactions that drive syn oligomerization (Figures 2 and 5). Moreover, binding of **CLXP1** to the N-terminal “anchor” region may also counteract membrane association of preformed syn aggregates, similarly to cholesterol derivatives, such as squalamine and trodusquemine, which compete with syn oligomers for lipid-binding sites.^111^ Consistent with these observations, **CLXP1**-mediated upregulation of the gene encoding lipid-dependent chaperone Hsp12 (Figure S5A) may further contribute to these protective effects (see above). Overall, interaction of **CLXP1** with the “anchor” region provides a mechanistic explanation for the observed dose-dependent reduction in intracellular foci formation (Figure 2A,B) and LD accumulation promoted by this anionic calixarene (Figure S5B).

Other residues with strong CSP values (Thr44 and His50; Figure 4B) are instead located in the pre-NAC portion of the N-terminal domain, which has been reported to function as a “sensor” modulating lipid-binding affinity^81^ (Figure 5). This region appears to be less tightly associated with membranes than the “anchor” region and exists in equilibrium between bound and unbound conformations.^8,81,109^ Within this region, a β-hairpin structure has been observed to form intramolecularly in monomeric syn, involving antiparallel β-strands spanning residues 37–43 and 48–54, connected by a β-turn (residues 44–47; Figure 5).^112^ This motif, stabilized by key residues such as His50 and Thr44,^112,113^ has been proposed to act as a nucleation center driving the pathological conversion of monomeric syn into oligomers.^16,112,114,115^ Several known inhibitors of syn aggregation, including β-wrapin AS69 ^113^ and phthalocyanine tetrasulfonate (PcTS),^116^ indeed exert their antiaggregant action by targeting residues within the β-hairpin region.

Based on our data, we propose that the interaction of **CLXP1** with the pre-NAC “sensor” region may further counteract the formation of toxic syn oligomers (Figure 2D), even when syn remains membrane-bound, by preventing the intermolecular contacts required for syn nucleation. This mechanism would favor retention of syn at the plasma membrane in a nontoxic α-helical conformation (Figures 2A and 5).

Overall, these findings suggest that binding of **CLXP1** to the N-terminal domain of syn modulates its conformational dynamics and aggregation propensity.

## Conclusions

In summary, our study identifies the phosphonatocalix[4]­arene **CLXP1** as a promising supramolecular modulator of syn-proteotoxicity. **CLXP1** counteracts the formation of toxic oligomeric species (Figure 2D) and intracellular inclusions composed of syn aggregates and lipid vesicle clusters, promoting syn localization at the plasma membrane (Figures 2A and 5). At the cellular level, **CLXP1** promotes yeast viability by preserving mitochondrial integrity and lipid homeostasis, mitigating oxidative stress, and enhancing autophagic clearance (Figures 3 and S5). **CLXP1** also appears to disrupt pathological feedback loops linking oxidative stress and lipid dysregulation to syn aggregation *in vivo* (Figure 5). NMR data reveal that **CLXP1** preferentially targets the N-terminal domain of syn (Figure 4), and this interaction may interfere with the conformational transitions required for its membrane binding and aggregation.

It has been proposed that membranes may facilitate syn aggregation by stabilizing monomeric species through the binding of the N-terminal “anchor” region, increasing the local concentration of syn and promoting pathogenic intermolecular interactions (Figure 5).^8,81,109^ In this context, the pre-NAC “sensor” region of the N-terminal domain appears to be less tightly associated with membranes.^8,81,109^ Within this more flexible region, the formation of an intramolecular β-hairpin has been proposed to provide a structural scaffold that promotes hydrophobic interactions between NAC domains of different monomers, thereby favoring the nucleation of oligomeric assemblies (Figure 5).^16,112,114,115^ Interestingly, similar β-hairpin motifs also appear to be implicated in the aggregation of β-amyloid (Aβ) peptides,^117,118^ suggesting a conserved structural trigger for amyloidogenic protein aggregation and a potential target for broad-spectrum antiamyloid strategies.

The interaction of **CLXP1** with the N-terminal domain of syn may thus provide a mechanistic basis for understanding calix[4]­arene-mediated modulation of the early steps of syn-membrane interactions (Figure 2A), the reduction of syn oligomer formation (Figure 2D), and the inhibition of intracellular foci coalescence (Figure 2A–C).

Altogether, these findings support the application of supramolecular host–guest chemistry to selectively interfere with the earliest aggregation events of intrinsically disordered proteins.^61,119^ Moreover, given the complexity of recapitulating the physiological interplay between syn and biological membranes *in vitro*, our study also supports the use of the yeast model of PD as a powerful and informative platform for dissecting the cellular mechanisms underlying neurodegeneration. While further validation in mammalian models is required, our results provide a strong foundation for the rational design of supramolecular scaffolds targeting syn and related amyloidogenic proteins.

## Supplementary Material



## Notes

Additional figures, materials, methods, and experimental details, including ^1^H, ^13^C, and ^31^P NMR spectra of all novel calix[4]­arene compounds (PDF)
