# Supplementary material for: A Rigid Supramolecular Solution to a Flexible Problem: A Multifunctional Calix[4]arene-Based Strategy to Prevent α‑Synuclein Toxicity
Source: ACS Cent Sci. 2026 May 4;12(5):669–83. doi: 10.1021/acscentsci.5c02416 (PMC13220195; doi:10.1021/acscentsci.5c02416)
Supplement: Supplementary file 1 [file oc5c02416_si_001.pdf]

Supporting Information

A rigid supramolecular solution to a flexible problem: a multifunctional calix[4]arene-based strategy to prevent α-synuclein toxicity

Davide Dell'Accantera,[1] Giulia Piccinini,[1] Cristina Ciabini,[2] Isabella C. Felli,[2] Stefano Volpi,[1] Nelson Marmiroli,[3] Francesco Sansone,[1] Roberta Ruotolo[1]

[1]Department of Chemistry, Life Sciences and Environmental Sustainability, University of Parma, Parco Area delle Scienze 11/A, 43124 Parma, Italy

[2]Magnetic Resonance Center (CERM) and Department of Chemistry "Ugo Schiff", University of Florence, Via L. Sacconi 6, 50019 Sesto Fiorentino, Italy

[3]Consorzio Interuniversitario Nazionale per le Scienze Ambientali (CINSA), University of Parma, Parco Area delle Scienze 11/A, 43124, Italy

Contents

Materials and Methods	pp. S2-S12
Supporting Figures S1-S10	pp. S13-S20
NMR spectra	pp. S21-S25
Supporting References	p. S26

Materials and Methods

Synthesis of anionic calix[4]arenes

All moisture sensitive reactions were carried out under nitrogen or argon atmosphere, using previously degassed solvents. Solvents and reagents obtained from commercial sources were used without further purification. Analytical TLC were performed using prepared plates of silica gel (Merck 60 F 254 on aluminum, Rahway, NJ, USA) and then revealed with UV lights. Merck silica gel 60 was used for flash chromatography (40–63 μm). ^{1}H-, ^{13}C- and 31P-NMR spectra were recorded on a Bruker AV400 spectrometer (400, 100 and 162 MHz as resonance frequency for ^{1}H, ^{13}C and ^{31}P respectively) and partially deuterated solvents were used as internal standards (δ values in ppm). All ^{13}C-NMR spectra were performed with proton decoupling. Electrospray ionization (ESI) mass analyses were performed with a Waters single-quadrupole spectrometer and a LTQ Orbitrap XL spectrometer in positive mode using MeOH as solvent. Flash chromatography was performed with a TeledyneIsco CombiFlash® NextGen using cartridge purchased by Sepachrom (Daily Standard Flash Cartridge Silica 60Å 50mm). Compounds **1**,[1] **3**,[2] 25,26,27,28-bis-crown-3-calix[4]arene[3] and **8**[4] were synthetized according to the procedures reported in literature. No unexpected or unusually high safety hazards were encountered.

Synthesis of 5,11,17,23-tetrakis((diethoxy)phosphonoyl)methyl-25,26,27,28-tetrahydroxycalix[4]arene (compound 2)

To a solution of compound **1** (1.15 g, 1.85 mmol, 1.0 equiv) in acetone (10 mL), NaI (1.67 g, 11.1 mmol, 6.0 equiv) and triethyl phosphite (1.43 mL, 8.36 mmol, 4.5 equiv) were added. The solution was kept stirring at room temperature and the reaction progress was monitored by TLC (eluent: dichloromethane/methanol 9/1). After 16 hours, the solvent was evaporated, the crude dissolved in dichloromethane (25 mL) and washed with water (25 mL) and brine (25 mL). Organic layer was collected and dried with anhydrous Na_2SO_4. The solvent was removed under reduced pressure and crude was purified by flash column chromatography (eluent: dichloromethane to dichloromethane/methanol 9/1) to obtain product as an oil (1.42 g, 74%). ^{1}H NMR ($CDCl_3$, 400 MHz): δ 10.07 (s, 4H, OH), 6.99 (s, 8H, ArH), 4.21 (br s, 4H, ArCH_{ax}HAr), 4.11 – 3.84 (m, 16H, $CH_2PO(OCH_2CH_3)_2$), 3.50 (br s, 4H, ArCHH_{eq}Ar), 2.92 (d, J = 21.2 Hz, 8H, $CH_2PO(OCH_2CH_3)_2$), 1.24 (t, J = 7.1 Hz, 24H, $CH_2PO(OCH_2CH_3)_2$). The spectroscopic data found are in agreement with those reported in literature.[5]

Synthesis of 5,11,17,23-tetrakis(phosphonoyl)methyl-25,26,27,28-tetrahydroxycalix[4]arene sodium salt (CLXP1)

A suspension of compound **2** (0.7 g, 0.68 mmol) in 37% HCl solution (10 mL) was kept stirring at reflux and the reaction progress was monitored by ESI-MS. After 4 days, the solvent was evaporated and the crude dissolved in water (5 mL). Through the treatment with NaOH the product was obtained as dark purple powder (0.45 g, 68%). ^{1}H NMR (D_2O, 400 MHz): δ 6.92 (d, J = 2.2 Hz, 8H, ArH), 4.45

– 3.76 (br s, 4H, ArCH_{ax}HAr), 3.75 – 3.03 (br s, 4H, ArCH_{eq}HAr), 2.48 (d, J = 19.3 Hz, 8H, CH_2P). The spectroscopic data found are in agreement with those reported in literature.[5]

Synthesis of 5,11,17,23-tetraformyl-25,26,27,28-tetra(ethoxycarbonylmethoxy)calix[4]arene (compound 4)

Tetraformylcalix[4]arene **3** (1.0 g, 1.86 mmol) and Na_2CO_3 (2.0 g, 18.7 mmol) were suspended in dry acetonitrile (28 mL) and suspension was kept stirring at reflux for 30 minutes. Then, ethyl bromoacetate (3.1 mL, 28.0 mmol) was added and the suspension was kept stirring at reflux. The reaction progress was monitored by TLC (eluent: dichloromethane/methanol 95/5). After 2 days, the solvent was evaporated and the crude dissolved in dichloromethane (30 mL) and washed with 1M HCl (30 mL). The aqueous phase was extracted with dichloromethane (15 mL) and the organic layers were collected and washed with water (30 mL) and brine (30 mL). The organic phase was dried with anhydrous Na_2SO_4 and the solvent evaporated under reduced pressure. The crude was dissolved in the minimum amount of dichloromethane and diethyl ether until the product precipitation as a slightly yellow powder (0.98 g, 60%). ^{1}H NMR ($CDCl_3$, 400 MHz): δ 9.63 (s, 4H, C*H*O), 7.22 (s, 8H, Ar*H*), 5.01 (d, *J* = 14.1 Hz, 4H, ArCH_{ax}HAr), 4.79 (s, 8H, CH_2COOCH$_2$CH$_3$), 4.25 (q, *J* = 7.2 Hz, 8H, CH$_2$COOCH_2CH$_3$), 3.47 (d, *J* = 14.2 Hz, 4H, ArCHH_{eq}Ar), 1.32 (t, *J* = 7.1 Hz, 12H,CH$_2$COOCH$_2$CH_3). ^{13}C NMR ($CDCl_3$,101 MHz): δ 191.1 (CHO), 169.3 (CH$_2$*C*OOCH$_2$CH$_3$), 161.0 (Ar), 135.2 (Ar), 132.1 (Ar), 130.5 (Ar), 71.4 (*C*H$_2$COOCH$_2$CH$_3$), 61.1 (CH$_2$COO*C*H$_2$CH$_3$), 31.4 (Ar*C*H$_2$Ar), 14.2 (CH$_2$COOCH$_2$*C*H$_3$). MS (ESI, m/z): 903.49 ([M+Na]$^+$).

Synthesis of 5,11,17,23-tetra(hydroxymethyl)-25,26,27,28-tetra(ethoxycarbonylmethoxy)calix[4]arene (compound 5)

Compound **4** (0.54 g, 0.61 mmol) was dissolved in absolute ethanol (18 mL) and sodium borohydride (0.035 g, 0.91 mmol) was added. The solution was kept stirring at room temperature and monitored through TLC (eluent: dichloromethane/ethyl acetate 9/1). After 30 minutes, 1 M HCl (20 mL) was added to the suspension and extracted with ethyl acetate (2x30 mL). The organic layers were collected and dried with anhydrous Na_2SO_4. The solvent was evaporated under reduced pressure to obtain the product as a white powder (0.53 g, 0.55 mmol). ^{1}H NMR ($CDCl_3$, 400 MHz): δ 6.71 (s, 8H, ArH), 4.89 (d, *J* = 13.3 Hz, 4H, ArCH_{ax}HAr), 4.76 (s, 8H, CH_2COOCH$_2$CH$_3$), 4.41 – 4.33 (m, 8H, CH_2OH), 4.29 – 4.16 (m, 8H, CH$_2$COOCH_2CH$_3$), 3.26 (d, *J* = 13.5 Hz, 4H, ArCHH_{eq}Ar), 1.31 (t, *J* = 7.1 Hz, 2H, CH$_2$COOCH$_2$CH_3). ^{13}C NMR ($CDCl_3$,101 MHz): δ 170.1 (C=O), 155.3 (Ar), 135.5 (Ar), 134.5 (Ar), 127.4 (Ar), 71.3(*C*H$_2$COOCH$_2$CH$_3$), 64.7 (*C*H$_2$OH), 60.5 (CH$_2$COO*C*H$_2$CH$_3$), 31.5 (Ar*C*H$_2$Ar), 14.2 (CH$_2$COOCH$_2$*C*H$_3$). MS (ESI, m/z): 911.36 ([M + Na]$^+$).

Synthesis of 5,11,17,23-tetrakis((diethoxy)phosphonoyl)methyl-25,26,27,28-tetra(ethoxycarbonylmethoxy)calix[4]arene (compound 7)

Compound **5** (0.795 g, 0.89 mmol) was dissolved in dry dichloromethane (5 mL) and the solution was cooled at 0 °C through an ice-bath. Then, $SOCl_2$ (0.36 mL, 4.92 mmol) was added and the solution was kept stirring at 0 °C and monitored by TLC (eluent: dichloromethane/ethyl acetate 9/1).

After 45 minutes, the reaction was quenched by solvent evaporation under reduced pressure. The crude was used directly for the next step without purification. The crude was dissolved in acetone (2 mL) and NaI (0.74 g, 4.96 mmol) and triethyl phosphite (0.64 mL, 3.72 mmol) were added. The solution was kept stirring for 16 hours and monitored by TLC (eluent: dichloromethane/methanol 95:5). After reagent consumption, the solvent was evaporated and crude was purified by flash column chromatography (eluent: dichloromethane to dichloromethane/methanol 95/5) to obtain product as oil (0.30 g, 30%). ^{1}H NMR ($CDCl_3$, 400 MHz): δ 7.15 (d, 8H, J = 2.56 Hz, ArH), 4.46 (s, 8H, CH_2COOCH$_2$CH$_3$), 4.38 (q, J = 7.1 Hz, 8H, CH$_2$COOCH_2CH$_3$), 4.21 (d, J = 12.2 Hz, 4H, ArCH_{ax}HAr), 3.92 (m, 16H, CH$_2$PO(OCH_2CH$_3$)$_2$), 3.44 (d, J = 12.3 Hz, 4H, ArCHH_{eq}Ar), 3.00 (d, J = 21.4 Hz, 8H, CH_2PO(OCH$_2$CH$_3$)$_2$), 1.42 (t, J = 7.1 Hz, 15H, CH$_2$COOCH$_2$CH_3), 1.03 (t, J = 7.1 Hz, 24H, CH$_2$PO(OCH$_2$CH_3)$_2$).^{13}C NMR ($CDCl_3$,101 MHz): δ 171.2 (C=O), 151.1 (Ar), 134.9 (Ar), 130.9 (Ar), 130.1 (Ar), 73.4 (CH$_2$COOCH$_2$CH$_3$), 62.2 (CH$_2$COOCH$_2$CH$_3$) 62.2 (CH$_2$PO(OCH$_2$CH$_3$)$_2$), 32.8 (d, J=138.5, CH$_2$PO(OCH$_2$CH$_3$)$_2$), 29.5 (ArCH$_2$Ar), 16.1 (CH$_2$PO(OCH$_2$$CH_3$)$_2$),14.2 (CH$_2$COOCH$_2$$CH_3$). ^{31}P NMR ($CDCl_3$, 162 MHz): δ 26.1. MS (ESI, m/z): 1391.53 ([M + Na]$^+$).

Synthesis of 5,11,17,23-tetrakis(phosphonoyl)methyl-25,26,27,28-tetra(hydroxycarbonylmethoxy)calix[4]arene sodium salt (CLXP2)

To a suspension of compound **6** (0.10 g, 0.07 mmol) in 1,4-dioxane (10 mL) was added 37% HCl solution (1 mL) and was kept stirring at reflux. The reaction progress was monitored by ESI-MS. After 16 hours, the solvent was evaporated and the crude dissolved in water (5 mL). Through the treatment with NaOH the product was obtained as a yellow powder (0.094 g, 99%). ^{1}H NMR (D_2O, 600 MHz): δ 6.98 (s, 8H, ArH), 4.36 (d, J = 12.4 Hz, 4H, ArCH_{ax}HAr), 4.25 (s, 8H, CH_2COONa), 3.30 (d, J = 12.5 Hz, 4H, ArCHH_{eq}Ar), 2.45 (d, J = 19.4 Hz, 8H, CH_2P). ^{13}C NMR ($CDCl_3$,101 MHz): δ 176.4 (C=O), 168.1 (Ar), 150.1 (Ar), 135.3 (Ar), 129.9 (Ar), 75.0 (CH$_2$COONa), 36.6 (CH$_2$PO$_3$Na$_2$), 29.6 (ArCH$_2$Ar). ^{31}P NMR ($CDCl_3$, 162 MHz): δ 20.4. MS (ESI, m/z): 1054.76 ([M-7Na+7H]$^+$).

Synthesis of 5,11,17,23-tetrakis(sulfonate)-25,26,27,28-bis-crown-3-calix[4]arene (CLXS3)

Under a nitrogen atmosphere, HSO_3Cl (0.5 mL) was cooled in a flask using an ice bath. 25,26,27,28-bis-crown-3-calix[4]arene (0.10 g, 0.27 mmol) was dissolved in dry dichloromethane (2.5 mL) and added dropwise to HSO_3Cl over the course of an hour. The reaction mixture was brought to room temperature and stirred for 6 hours and monitored by ESI-MS, after which the reaction was quenched by adding ice (as needed). A viscous brown solid precipitated. The solid was separated by filtration from the liquid phase, dried, triturated with MeOH/DCM 1:1, filtered, and dried again, yielding the desired compound as an opaque white solid. (0.081 g, 34%). ^{1}H NMR (DMSO-d_6, 400 MHz): δ 7.26 (m, 8H, ArH), 4.98 (d, J = 11.9 Hz, 2H, ArCH_{ax}HAr), 4.44 (d, J = 12.0 Hz, 2H, ArCH_{ax}HAr), 4.34 – 4.15 (m, 12H, OCH$_2$CH$_2$O), 3.66 (dt, J = 11.6, 6.1 Hz, 4H, ArOCHHCH$_2$O), 3.41 (d, J = 12.2 Hz, 2H, ArCH_{eq}HAr), 3.34 (d, J = 11.9 Hz, 2H, ArCH_{eq}HAr).

The spectroscopic data found are in agreement with those reported in literature.[6]

Synthesis of 5,11,17,23-tetrakis(phosphonoyl)methyl-25,26,27,28-tetra(hydroxycarbonylmethoxy)calix[4]arene sodium salt (CLXP4)

A solution of compound **8** (0.30 g, 0.45 mmol) in tris(trimethylsilyl)phosphite (3 mL) was kept stirring in Schlenk at 80 °C and the reaction progress was monitored by ESI-MS. After 16 hours, the unreacted tris(trimethylsilyl)phosphite was removed by distillation under vacuum. The crude was dissolved in methanol (3 mL) and kept stirring for 16 hours at room temperature. Methanol was evaporated under reduced pressure and through the treatment with NaOH the product was obtained as a yellow powder (0.350 g, 77%). Here we reported the signal of the largely most abundant 1,3-alternate conformation. ^{1}H NMR (D_2O, 400 MHz): δ7.03 (s, 8H, ArH), 3.70 (br s, 8H, ArCH_2Ar), 3.37 (br s, 12H, OCH_3), 2.66 (d, J = 19.1 Hz, 8H, CH_2P). ^{13}C NMR (D_2O,101 MHz): δ 161.9 (Ar), 154.7 (Ar), 134.5 (Ar), 130.6 (Ar), 58.6 (OCH_3), 36.5 (d, $J_{C\text{-}P}$ = 120 Hz, *C*H2P), 35.9 (ArCH_2Ar).^{31}P NMR (D_2O, 162 MHz): δ 18.2 MS. (ESI, m/z): 855.3 ([M-H]$^-$).

Yeast strains and growth conditions

The HiTox strain (W303 *pdr1Δ pdr3Δ* genetic background) carrying two copies of the syn-GFP gene integrated into the *URA3* and *TRP1* loci was used as a model of Parkinson's Disease (PD).[7] The expression of syn fused at its C-terminus to GFP was driven by the galactose-inducible *GAL1* promoter.

Cells were cultured at 28 °C in synthetic defined minimal medium containing 0.67% (w/v) yeast nitrogen base without amino acids, supplemented with adenine (20 µg/mL), histidine (20 µg/mL), leucine (30 µg/mL), and either 2% (w/v) glucose (repressive condition; SD medium) or 2% (w/v) galactose (inducing condition; SGal medium) for the indicated times.

For mitochondrial morphology analysis, HiTox cells transformed with the pYX142 plasmid (*LEU2* selectable marker), harboring the gene encoding a mitochondria-localized red fluorescent protein (mtRFP), were used.[8]

Fluorescence microscopy

Images were acquired using a Zeiss Axio Imager.Z2 fluorescence microscope (Carl Zeiss Microscopy GmbH) with the same exposure settings to ensure reproducibility. At least 150 cells were analyzed per condition from three independent experiments.

To analyze syn-GFP cellular localization, overnight pre-cultures grown in SD medium were harvested, washed twice with sterile Milli-Q water, and inoculated into SGal medium at an initial optical density value at 600 nm (OD_{600}) of 0.05. To assess the protective effects of calix[4]arenes on syn-overexpressing yeast during chronological aging,[8,9] cells were incubated in 96-well plates at 28 °C for 48 h without agitation with or without calix[4]arenes (at various concentrations as indicated in the text). After treatment, syn-overexpressing cells were imaged using a fluorescence microscope. The percentage of GFP-positive (GFP$^+$) cells was calculated relative to total cell number; the percentage of cells exhibiting intracellular (small or large) foci or lacking foci was quantified relative to the GFP$^+$ population.

Propidium iodide staining

Cell viability was assessed by staining with propidium iodide (PI; Sigma-Aldrich, Merck), a membrane-impermeant DNA dye excluded from viable cells. Yeast cells cultured in SGal medium for 48 h were collected, washed with sterile Milli-Q water, and stained with PI (2 μM, final concentration) for 30 min. After washing with PBS, cells were imaged using a fluorescence microscope (excitation/emission: 536/617 nm).

Clonogenic survival assay

Yeast cells (starting from an OD_{600} value of 0.05) were grown in SGal medium at 28 °C for 48 h in 96-well plates without agitation in the presence or absence of CLXP1 (10 μM, final concentration). To assess cell viability following syn overexpression, cultures were diluted 1:500, and plated onto SD agar (non-inducing conditions). Colony-forming units (CFU) were counted after 72 h of incubation at 28°C.[8]

Protein extraction

Yeast cells (starting from an OD_{600} value of 0.2) were grown in SGal medium at 28 °C for 24 h with shaking in the presence or absence of **CLXP1** (10 μM, final concentration). After treatment, cells were harvested by centrifugation, washed with sterile Milli-Q water, snap-frozen in liquid nitrogen, and stored at -80 °C. Cell lysis was performed in ice-cold buffer [25 mM Tris-HCl pH 7.5, 50 mM KCl, 1 mM $MgCl_2$, 1 mM EDTA, 1% Triton X-100 (v/v), 10% glycerol (v/v), and protease inhibitors] in the presence of glass beads (0.5 mm diameter), using a Mini-Beadbeater-16 (BioSpec Products Inc.); five cycles of bead-beating (1 min stroke followed by 5 min incubation on ice) were performed. Lysates were clarified by centrifugation (1000 rpm for 10 seconds at 4 °C), and the protein concentration of whole cell extracts was determined using the Bradford assay (Bio-Rad) according to the manufacturer's instructions.

Dot blot assay

Dot blot analysis was performed in a 96-well format using a Bio-Dot microfiltration apparatus (Bio-Rad) for vacuum-transfer of protein samples (whole cell extracts; 50 μg of total protein per sample) onto nitrocellulose membranes (0.2 μm pore size; Bio-Rad) prewetted with Tris-buffered saline [TBS; 20 mM Tris-HCl pH 7.4, 0.8% NaCl (w/v)]. After sample loading, membranes were blocked for 2 h at room temperature in TTBS buffer [TBS supplemented with 0.1% Tween 20 (v/v)] containing 5% BSA (w/v), and incubated overnight at 4 °C with the following primary antibodies: anti-syn-aggregate (MJFR14-6-4-2, Abcam; 1:20000 dilution), anti-syn (Santa Cruz; 1:1000 dilution), and anti-PGK1 (Abcam; 1:2000 dilution). Anti-PGK1 antibody was used as a loading control. After washing with TTBS, membranes were incubated for 1 h at room temperature with IRDye-labeled goat anti-rabbit (for MJFR14-6-4-2) or goat anti-mouse (for anti-PGK1 and anti-syn) secondary antibodies (LI-COR Biosciences; 1:15000 dilution). After washing with TTBS, membranes were dried, and signals were detected using a ChemiDoc MP Imaging System (Bio-Rad).

Oxidative stress detection

Oxidative stress was assessed using CellROX™ Orange Reagent (Thermo Fisher Scientific), a non-fluorescent dye in its reduced state that exhibits bright orange fluorescence upon oxidation by reactive oxygen species (ROS). Yeast cells (starting from an OD_{600} value of 0.2) were grown in SGal medium for 4 h at 28 °C with agitation in the presence or absence of **CLXP1** (1-10 µM). Cells were then stained with CellROX™ Orange Reagent (Thermo Fisher Scientific; 5 µM, final concentration) for 30 min at 37 °C. After washing with PBS, cells were imaged using a fluorescence microscope (excitation/emission at 545/565 nm).

ABTS radical scavenging assay

Antioxidant activity was measured using the 2,2'-azino-bis(3-ethylbenzothiazoline-6-sulphonic acid) (ABTS; Sigma-Aldrich, Merck) assay. This colorimetric assay evaluates the ability of antioxidants to reduce the dark blue ABTS radical cation ($ABTS^{\bullet+}$) to its colorless form (ABTS). $ABTS^{\bullet+}$ was generated by mixing 7 mM ABTS with 2.45 mM potassium persulfate (1:1, v/v) and incubating the solution overnight at 4 °C in the dark. The resulting solution was diluted in ethanol to an absorbance of approximately 0.7 at 724 nm (OD_{724}). Whole cell extracts (20 µg of total protein per sample) obtained from cells grown in SGal medium for 24 h at 28 °C in the presence or absence of **CLXP1** (10 µM, final concentration) were incubated with 1 mL of the $ABTS^{\bullet+}$ solution for 30 min at room temperature, and OD_{724} was measured.

DCFDA assay

Oxidative stress was also assessed using 2',7'-dichlorofluorescin diacetate (DCFDA; Sigma-Aldrich, Merck), a vital dye for detecting intracellular reactive oxygen species (ROS). Yeast cells (starting from an OD_{600} value of 0.001) were grown in SD medium (repressing conditions) at 28 °C with shaking until reaching an OD_{600} value of 0.5. Cells were then treated with **CLXP1** (10 µM, final concentration) or **CLXP3** (10 µM, final concentration) for 4 h at 28 °C with shaking. Control samples without calixarene treatments were included. Oxidative stress was subsequently induced by adding tert-butyl hydroperoxide (tBHP; Sigma-Aldrich, Merck) to a final concentration of 1 mM for 15 min. Cells were then incubated in the dark with DCFDA (10 µM, final concentration) for 30 min at 37 °C, washed with PBS, and analyzed using a SpectraFluor microplate reader (Tecan; excitation/emission 492/525 nm).

Mitochondrial morphology analysis

HiTox cells transformed with the mtRFP-pYX142 plasmid were used to assess mitochondrial morphology. Cells (starting from an OD_{600} value of 0.2) were grown in SGal medium at 28 °C for 24 h with agitation in the presence or absence of **CLXP1** (10 µM, final concentration). Cells were then imaged using a fluorescence microscope (TRITC filter). Mitochondria were classified as "tubular" or "fragmented" based on RFP fluorescence.

Lipid droplet staining

Cells (starting from an OD_{600} value of 0.2) were grown in SGal medium at 28 °C for 4 h with agitation in the presence or absence of **CLXP1** (10 µM, final concentration). Cells were then stained with Nile Red dye (Sigma-Aldrich, Merck; 4 µg/mL, final concentration) for 30 min at 37 °C, washed with PBS and imaged using a fluorescence microscope (excitation/emission: 552/636 nm).

GAL1 promoter shut-off experiments

Cells (starting from an OD_{600} value of 0.2) were grown under inducing conditions (SGal medium) at 28 °C for 24 h in the presence or absence of **CLXP1** (1-10 µM). Cells were harvested by centrifugation, washed twice with sterile water, and transferred to repressing (glucose-containing) medium to shut off *GAL1* promoter activity. Cultures were further incubated at 28 °C with agitation for the indicated time points. The percentage of fluorescent (GFP^+) cells displaying syn foci was quantified at 0, 2, and 8 h post-transfer, and values were normalized to the initial time point (0 h).

Gene expression analysis

Cells (starting from an OD_{600} value of 0.2) were grown in SGal medium at 28 °C for 6 h with shaking in presence of **CLXP1** treatment (10 µM, final concentration). Parallel untreated samples were included as controls. After treatment, cells were collected and stored at -80 °C. Total RNA extraction was performed using the RNeasy Mini Kit (Qiagen), according to the manufacturer's instructions. RNA samples were then quantified using a NanoDrop™ 2000/2000c (Thermo Fisher Scientific). Total RNA (1 µg) was reverse-transcribed using QuantiTect® Reverse Transcription Kit (Qiagen), and cDNA was quantified by real-time PCR using the PowerUp SYBR™ Green Master Mix (Thermo Fisher Scientific) and an ABI PRISM 7000 Sequence Detection System (Applied Biosystems). Relative gene expression was quantified using the comparative C_T method, with *18S rRNA* as the housekeeping gene. PCR primer sequences used in this manuscript were indicated in **Table S1**.

Gene name	Forward primer sequence	Reverse primer sequence
ATG17	5'-CAGGGAACTAACGGACCTTG-3'	5'-TTCTTTGTCGTCGCCTTGTAC-3'
HSP12	5'-ACTCTGCCGAAAAAGGCAAGG-3'	5'-GACGGCATCGTTCAACTTGG-3'
RPN4	5'-GATTTCCATCTCCCTCAACCTC-3'	5'-CTAACGCAACTACGGTTACTGG-3'
18S rRNA	5'-CACCAGGTCCAGACACAATAAG-3'	5'-TCTCGTTCGTTATCGCAATTAAG-3'

Table S1. List of primers used in this study.

Lysine-CLXP1 interaction

The investigation of the complexation between **CLXP1** and lysine was carried out exploiting the dipeptide Nα-acetyl-L-lysyl-glycine-OMe as small model. In this peptide, the lysine is involved in two amide bonds simulating its condition in protein. The dipeptide was employed in its trifluoroacetate salt form, obtained after removal of the Boc protecting group with trifluoroacetic acid at the end of the synthesis.

NMR titration

The ^{1}H NMR titrations were performed in D_2O at room temperature, starting with a guest concentration of 1.5 mM, to which increasing aliquots of **CLXP1** were added from a 10 mM stock solution as reported in **Table S2**.

	added Vol (μl)	added total Vol (μl)	tot. Vol. (μl)	$[CLXP1]_0$ (mM)	$[Peptide]_0$ (mM)	$[CLXP1]_0/[Peptide]_0$
1	0	0	500	0.00	1.58	0.00
2	10	10	510	0.20	1.55	0.13
3	15	25	525	0.48	1.50	0.32
4	20	45	545	0.83	1.45	0.57
5	25	70	570	1.23	1.39	0.89
6	30	100	600	1.67	1.32	1.27
7	50	150	650	2.31	1.22	1.90
8	100	250	750	3.33	1.05	3.16

Table S2. Values of volume and concentration for every point of titration.

Representative spectra from these titrations are shown in **Figure S6**. A preliminary inspection revealed distinct chemical shift perturbations in the dipeptide resonances upon incremental addition of the host. This behavior indicates (i) a specific host–guest interaction and (ii) a binding process occurring in the fast exchange regime on the NMR timescale. The most pronounced perturbations were observed for the lysine side-chain protons. In particular, the methylene resonances of the side chain, appearing as multiplets between 1.2–1.8 ppm and as a triplet at 2.9 ppm, progressively shifted downfield and lost fine splitting, evolving from well-defined multiplets/triplets (traces 1–5) to broadened, singlet-like signals (traces ≥6). Notably, the signal at 2.9 ppm, corresponding to the CH_2 group adjacent to the terminal ammonium moiety, exhibited a substantial shift from 2.91 to 1.64 ppm. This pronounced change is consistent with literature precedents for lysine complexation by calixarene derivatives and is attributed to the inclusion of the lysine side chain within the macrocyclic cavity, accompanied by electrostatic interactions between the ammonium group and one or more phosphonate functionalities of the host.

Quantitative analysis of the titration data was performed using the online tool supramolecular.org. The software applies nonlinear regression (Nelder–Mead method) to generate a binding isotherm (solid line in **Figure S6**). From the chemical shift variation of the methylene protons adjacent to the ammonium group (CH_2–NH_3^+), an association constant (K_a) of 1517 ± 6 M^{-1} was determined.

ITC titration

For direct titrations, all experiments were conducted in a water solution maintained at atmospheric pressure and 25° C. A fixed volume of 2.00 μl per injection of the guest solution ranging from 2.00 to 5.00 millimolar was injected into the reaction cell with a 280 μl capacity. The reaction cell was

charged with either 200 μM of the **CLXP1** solution, and each injection was completed within 4 seconds under stirring at 750 rpm. A total of nineteen consecutive injections were made for each titration. The heat of dilution was gauged by introducing the guest (dipeptide) solution in water. The actual reaction heat was determined by deducting the dilution heat from the apparent reaction heat. The resulting net reaction heat was then processed through computer simulation employing the "One Set of Sites" model using the MicroCal PEAQ-ITC Analysis Software. The binding stoichiometry was held constant at 1. The association constants were determined from the reciprocal of the dissociation constants provided by the software.

As shown in **Figure S8**, the binding process is endothermic; nevertheless, the free energy change (ΔG) is negative, driven by a favourable entropic contribution. This entropic gain is consistent with a hydrophobic effect, arising from the encapsulation of the lipophilic portion of the lysine side chain within the macrocyclic cavity. Interestingly, the ITC fitting revealed an n value close to 0.5, suggesting that a second dipeptide unit may interact with the calixarene host under the conditions employed.

Syn-CLXP1 interaction

Protein production

^{15}N-labelled syn was expressed in *Escherichia coli* BL21 Gold (DE3) cells transformed with the plasmid pT 7-7 containing the sequence encoding human syn cDNA. Transformed cells were grown at 37 °C in ^{15}N-labelled M9 minimal medium. When the culture reached an OD_{600} value of 0.6, syn expression was induced with isopropyl-β-D-thiogalactopyranoside (IPTG; 1 mM, final concentration) at 37 °C for 4 h. Cells were harvested by centrifugation at 5000 rpm for 10 min and resuspended in lysis buffer (50 mM Tris-HCl pH 8, 5 mM EDTA, and protease inhibitors). Cells were lysed by sonication, and the lysate was clarified by ultracentrifugation at 35000 rpm for 30 min at 4 °C. The clarified supernatant was loaded onto a HiTrap Q FF 16/10 anionic exchange column equilibrated in 50 mM Tris-HCl pH 8, 5 mM EDTA. Bound protein was eluted using a linear NaCl gradient (0-1 M). The protein was further purified by size exclusion chromatography on a Superdex 26/60 gel filtration column using an elution buffer suitable for NMR experiments (25 mM HEPES pH 7.2, 25 mM KCl). Protein-containing fractions were pooled and concentrated.

Titration

The titration of syn with **CLXP1** was performed using ^{15}N-labelled protein (190 μM) in HEPES buffer (25 mM HEPES pH 7.2, 25 mM KCl). The interaction with **CLXP1** was monitored by recording 2D ^{1}H-^{15}N HSQC NMR spectra (hereafter referred to as 2D HN NMR spectra), starting from the reference spectrum of the free protein (without **CLXP1** supplementation). Subsequent spectra were acquired after the addition of increasing amounts of **CLXP1** to the protein sample. Titration points were selected based on the spectral changes observed of the protein at the following syn:**CLXP1** molar ratios: 1:0.0, 1:0.2, 1:0.6, 1:1.2, 1:2.4, 1:4.8, and 1:9.6. The pH value was monitored throughout the experiment.

NMR experiments

To follow the titration, 2D HN NMR spectra were acquired. All experiments were acquired on a Bruker AVANCE NEO spectrometer operating at 900.06 (^{1}H) and 91.20 (^{15}N) MHz equipped with a cryogenically cooled probehead (TCI). NMR measurements were performed at 298 K. Detailed acquisition parameters are reported in **Table S3**.

Experiment	Data points		Spectral width (ppm)		Number of scans	Relaxation delay (s)
	**F2**	**F1**	**F2**	**F1**		
2D ^{1}H^{15}N HSQC	2048	412	12.08	26.06	8	1
^{15}N R_1	2048	256	12.08	26.06	8	3.5
^{15}N R_2	2048	256	12.08	26.06	8	3.5

Table S3. Acquisition parameters for the recorded spectra.

^{15}N relaxation values (^{15}N R_1, ^{15}N R_2) were recorded using standard Bruker pulse sequences, with acquisition parameters reported in **Table S3**.
For the determination of ^{15}N R_1 values, the following delays were used: 41.00 ms, 80.00 ms, 160.00 ms, 320.00 ms, 520.00 ms, 760.00 ms, 1000.00 ms, 1200.00 ms and 1400.00 ms.
For the determination of ^{15}N R_2 values, the following delays were used: 44.48 ms, 88.96 ms, 133.44 ms, 177.92 ms, 266.88 ms, 355.84 ms, 444.80 ms and 533.76 ms.

NMR spectral analysis

All NMR spectra were acquired and processed using Bruker TopSpin 4.1.3 software. Chemical shifts were calibrated using the DSS as a standard. Spectral analysis was performed using CARA, utilizing the NEASY tool. Resonance assignments were taken from Tagliaferro et al.[10] To monitor chemical shift changes upon ligand binding, peak positions were manually adjusted for each titration point. This procedure enabled the determination of chemical shift perturbation (CSP) values, which were subsequently analyzed. CSP values were calculated according to the following equation:[11]

$$CSP = \sqrt{\frac{1}{2}\left[\delta_H^2 + (0.1\delta_N)^2\right]}$$

were δ_H and δ_N represent the variations of chemical shifts of the^{1}H and ^{15}N nuclear spins. From the variations of CSP values upon addition of increasing amounts of ligand, it was also possible to obtain and estimate the dissociation constant (K_d)[11] assuming a 1:1 interaction using the following equation:

$$\Delta_{obs} = \Delta_{max}\frac{C_P + C_L + K_d - \sqrt{(C_P + C_L + K_d)^2 - 4C_PC_L}}{2C_P}$$

where Δ_{obs} is the observed CSP at the different titration points, Δ_{max} is the maximum value obtained at the end of the titration, C_P is the total protein concentration, C_L is the ligand concentration at the different titration points, and K_d is the dissociation constant.

Statistical analysis

For each experiment, at least three biological replicates were performed. Data were reported as mean ± standard deviation (SD). Statistical analysis was performed using GraphPad Prism v10.5. Statistical significance was determined using one-way ANOVA, followed by Bonferroni's multiple comparisons test or two-tailed unpaired t-test (*, $p < 0.05$; **, $p < 0.01$; ***, $p < 0.001$; ****, $p < 0.0001$), as indicated in the text.

Supporting Figures

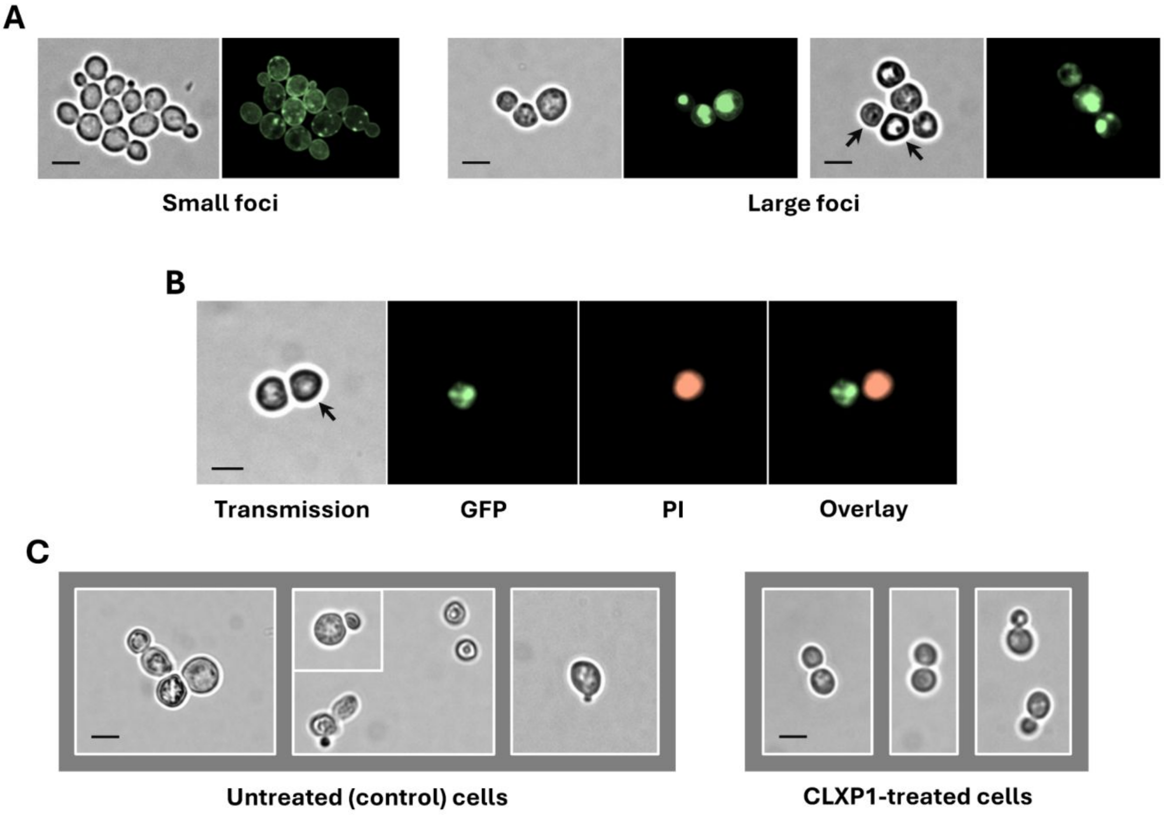

Figure S1. Effects of syn overexpression in the HiTox strain. A) Formation of small or large intracellular foci in GFP-positive (GFP^+; *green fluorescence*) syn-overexpressing cells. **B**) After 48 h at 28 °C under inducing conditions (SGal medium), dead cells are GFP-negative (GFP^-) and propidium iodide-positive ($PI^{+;}$ *red fluorescence*). Phase-contrast (*left*) and fluorescence (*right*) images are shown for each condition. Black arrows indicate inviable yeast cells. Scale bars: 5 µm. **C**) Untreated syn-overexpressing cells appear larger, rounder, and frequently exhibit aberrant buds, whereas **CLXP1**-treated cells display the ovoid morphology characteristic of healthy budding yeast.

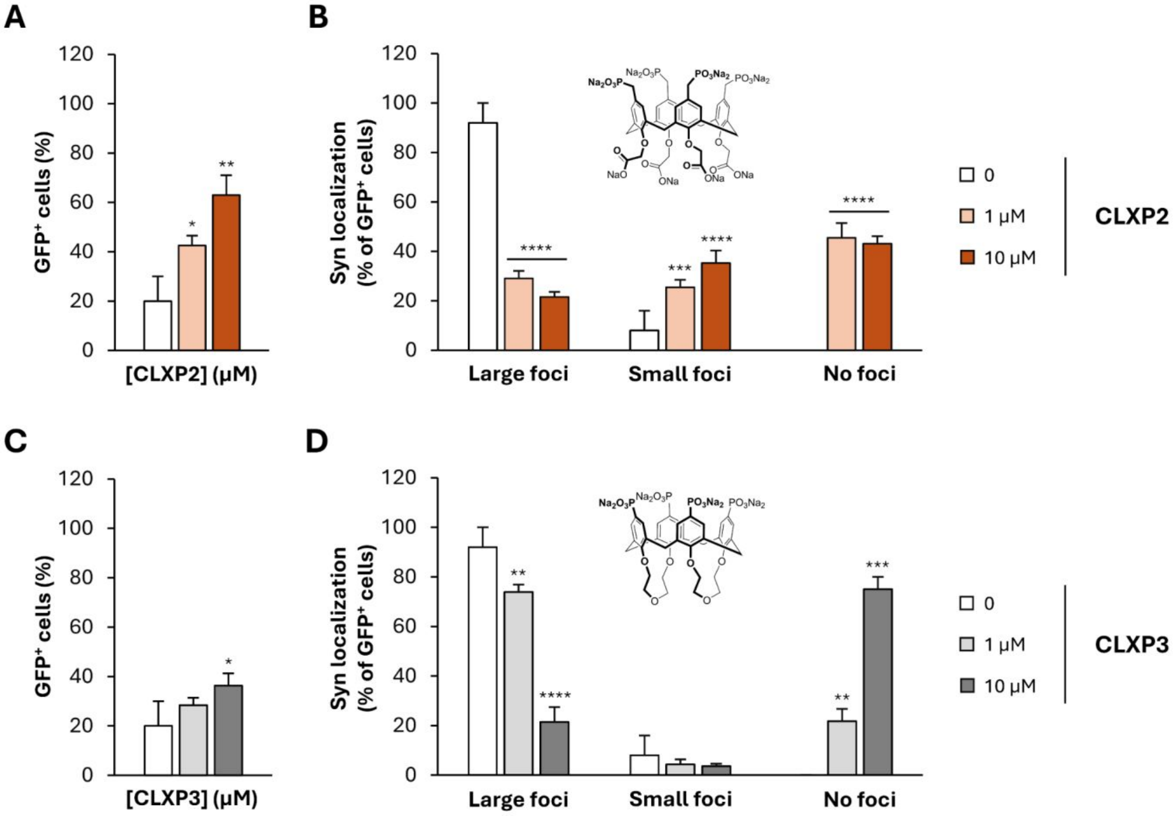

Figure S2. CLXP2 and CLXP3 modulate intracellular syn foci formation. Yeast cells were treated in the presence or absence of increasing concentrations (1-10 µM) of **CLXP2** (**A**, **B**) or **CLXP3** (**C**, **D**) for 48 h at 28 °C. The percentage of GFP-positive (GFP^+) cells relative to the total cell population was quantified (**A**, **C**). The percentage of GFP^+ cells displaying intracellular foci (large or small) or no detectable foci (with syn predominantly localized at the plasma membrane) was calculated relative to the total number of GFP^+ cells (**B**, **D**). Statistical significance was assessed by one-way ANOVA followed by Bonferroni's multiple comparisons test (*, $p < 0.05$; **, $p < 0.01$; ***, $p<0.001$; ****, $p < 0.0001$).

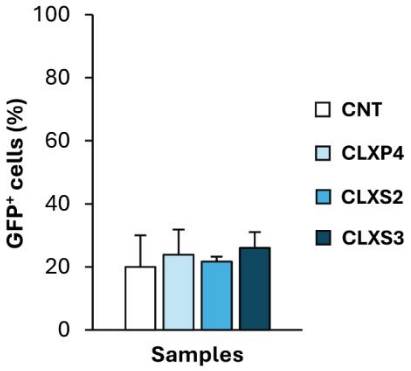

Figure S3. Effect of CLXP4, CLXS2, and CLXS3 treatment in syn-overexpressing cells. Yeast cells were treated with **CLXP4, CLXS2**, or **CLXS3** (10 μM, final concentration) for 48 h at 28 °C. Untreated cells were used as control sample (*CNT*). The percentage of GFP-positive (GFP^+) cells relative to the total cell population was quantified.

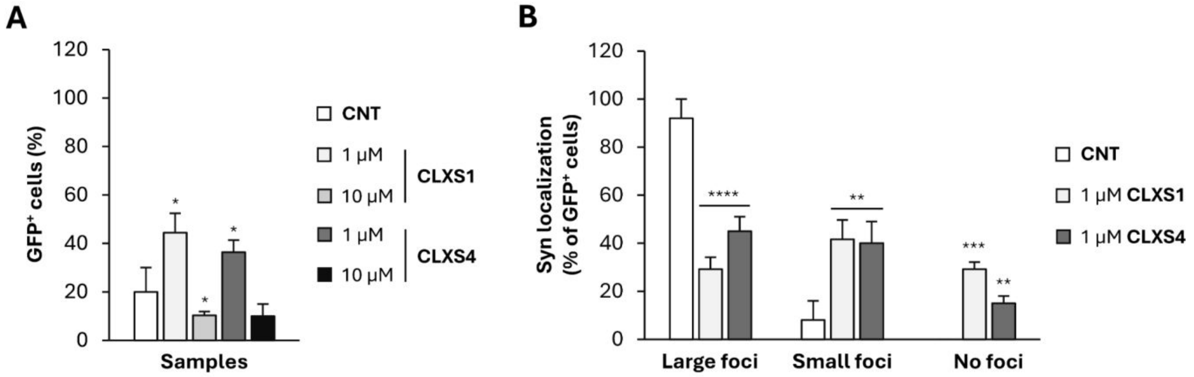

Figure S4. Effect of CLXS1 and CLXS4 treatment in syn-overexpressing cells. Yeast cells were treated with **CLXS1** or **CLXS4** (1-10 μM) for 48 h at 28 °C. Untreated cells were used as control sample (*CNT*). The percentage of GFP-positive (GFP^+) cells relative to the total cell population was quantified (**A**). The percentage of GFP^+ cells displaying intracellular foci (large or small) or no detectable foci (with syn predominantly localized at the plasma membrane) was calculated relative to the total number of GFP^+ cells (**B**). Statistical significance was assessed by one-way ANOVA followed by Bonferroni's multiple comparisons test (*, $p < 0.05$; **, $p < 0.01$; ***, $p<0.001$; ****, $p < 0.0001$).

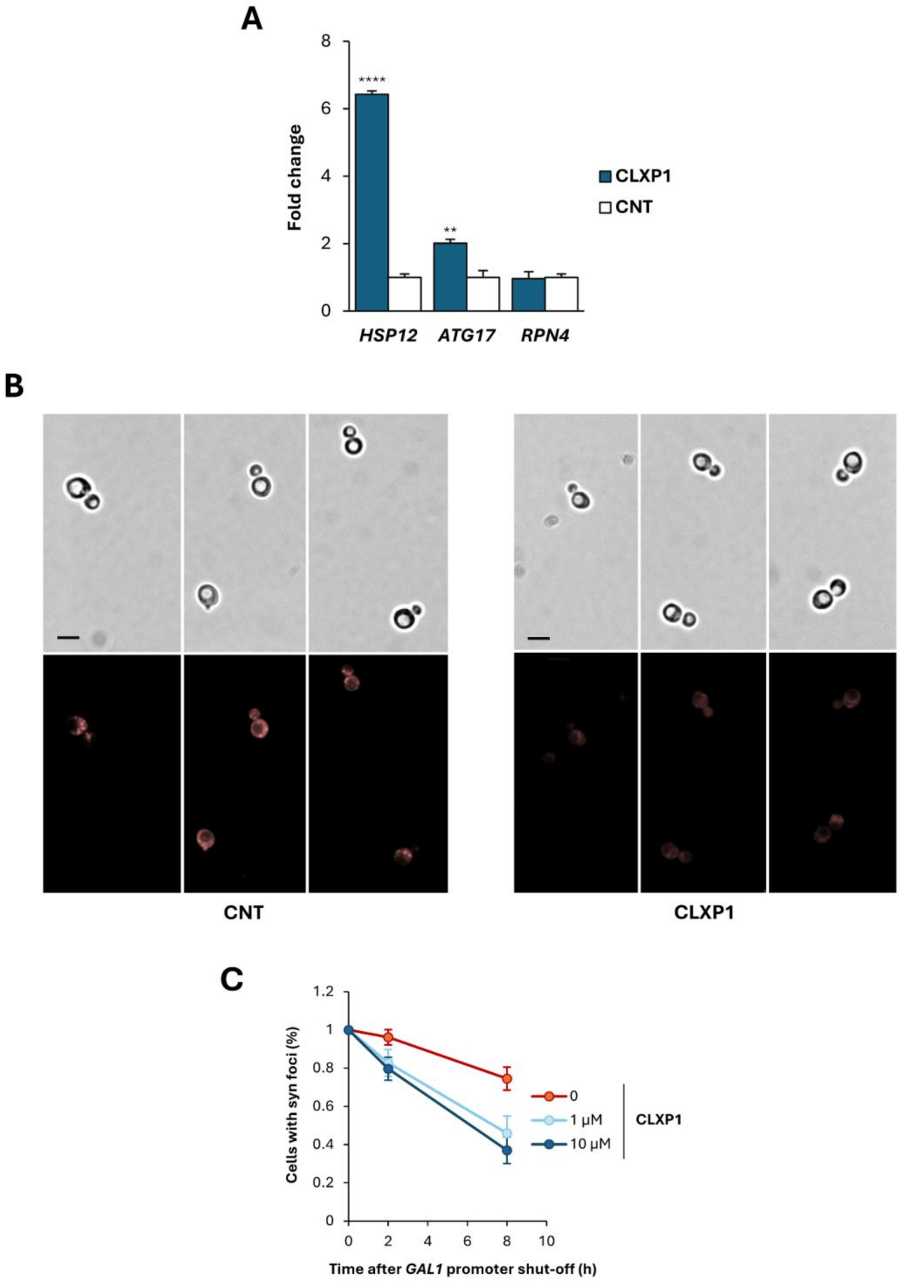

Figure S5. CLXP1 modulates lipid homeostasis and clearance pathways in the HiTox strain. A) Gene expression analysis by quantitative real-time PCR. Statistical analysis was performed using two-tailed unpaired t-test (**, $p < 0.01$; ****, $p < 0.0001$). **B**) **CLXP1** treatment decreases lipid droplet accumulation in syn-overexpressing cells. Yeast cells were grown in the absence (*CNT*) or presence of **CLXP1** (10 μM) for 4 h a 28 °C. Lipid droplets were stained using the Nile red fluorescent dye. For each condition, both phase-contrast (*top*) and fluorescence (*bottom*) images are shown. Scale bars: 5 μm. **C**) Clearance of syn foci following *GAL1* promoter shut-off in the HiTox strain. Cells were cultured for 24 h under inducing conditions (galactose-containing medium), harvested by centrifugation, washed twice with sterile water, and transferred to repressing (glucose-containing) medium to shut off expression from the *GAL1* promoter. The percentage of fluorescent cells displaying syn foci was quantified at 0, 2 and 8 h after transfer in repressing conditions and normalized to the initial time point (0 h).

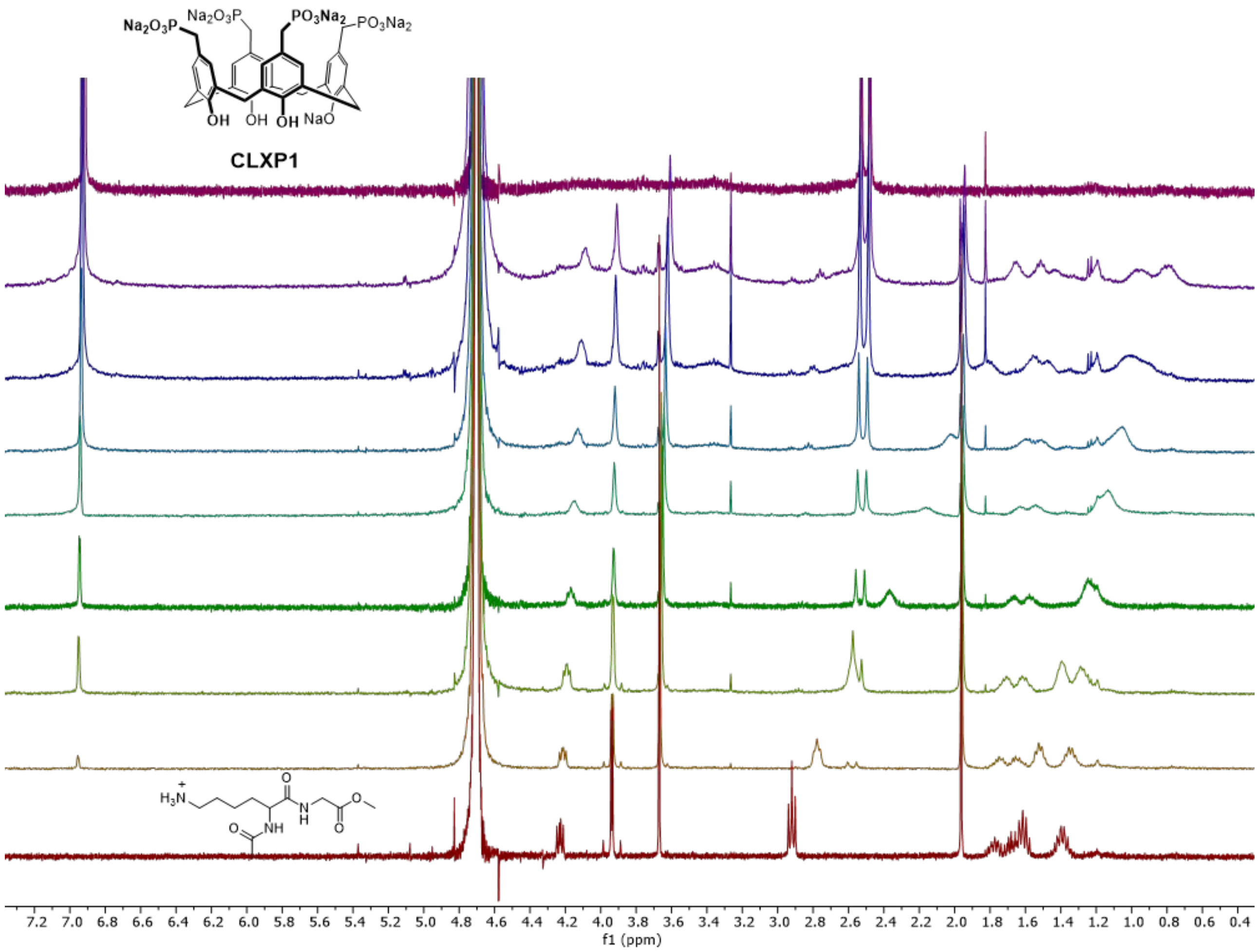

Figure S6. Spectra related to the ^{1}H NMR titration in D_2O (400 MHz, 298 K) of N-acetyl-L-lysyl-glycine methyl ester dipeptide (1.58 mM) with a solution of **CLXP1** 10 mM.

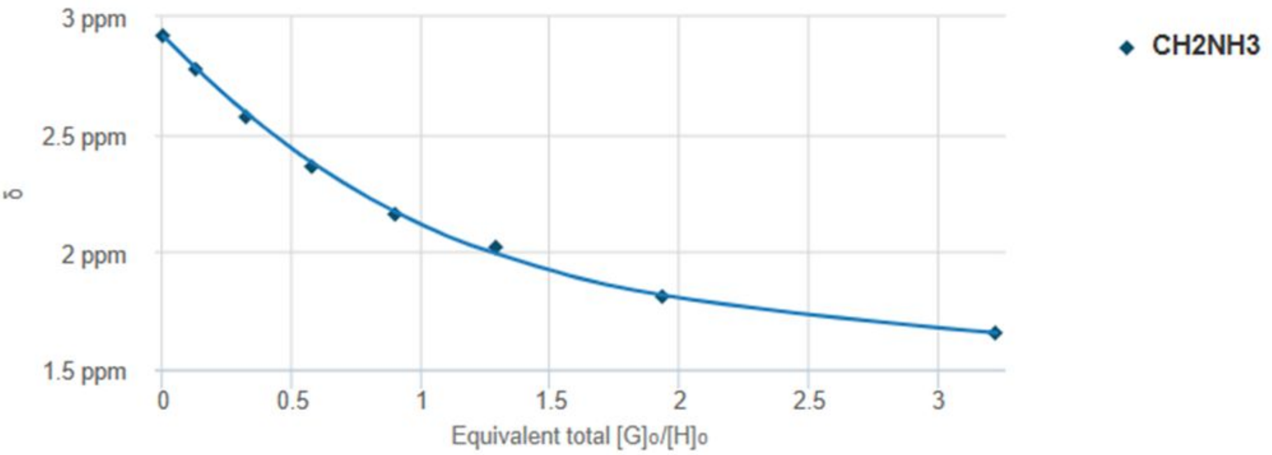

Figure S7. Experimental points and binding isotherm related to the shift of Lys CH_2N group during the titration of N-acetyl-L-lysyl-glycine methyl ester dipeptide with **CLXP1** in D_2O.

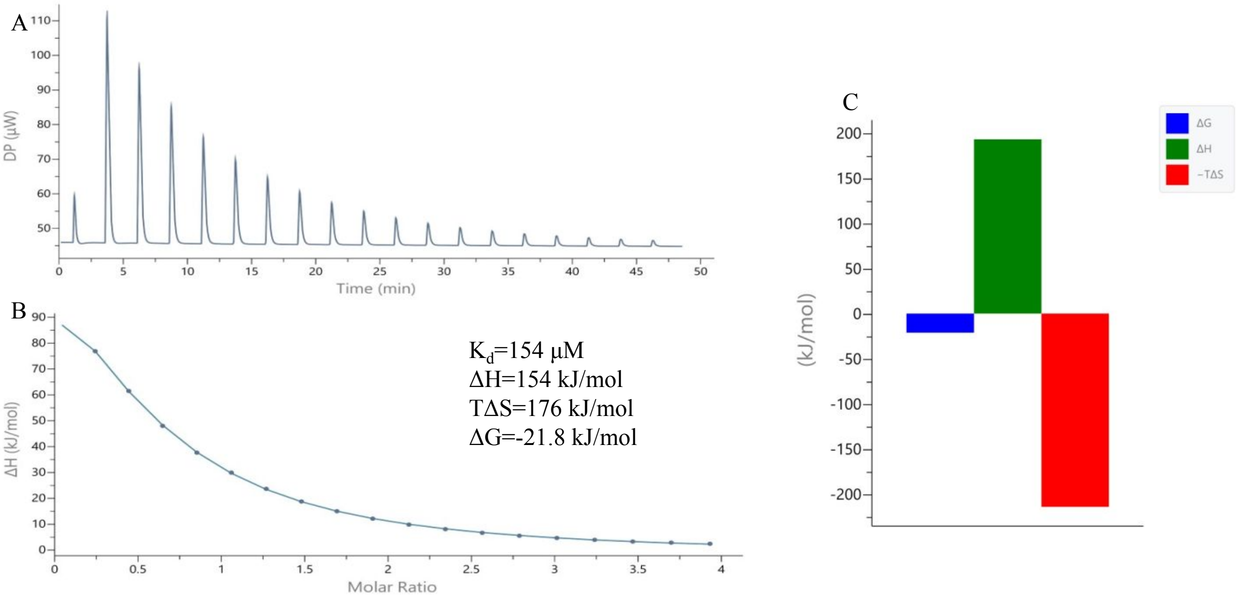

Figure S8. Isothermal titration calorimetry (ITC) analysis of N-acetyl-L-lysyl-glycine methyl ester binding to CLXP1. A) Raw data binding from ITC titration of N-acetyl-L-lysyl-glycine methyl ester dipeptide with **CLXP1**. **B**) Isothermal binding and thermodynamic values of titration. **C**) Graphical representation of thermodynamic parameters.

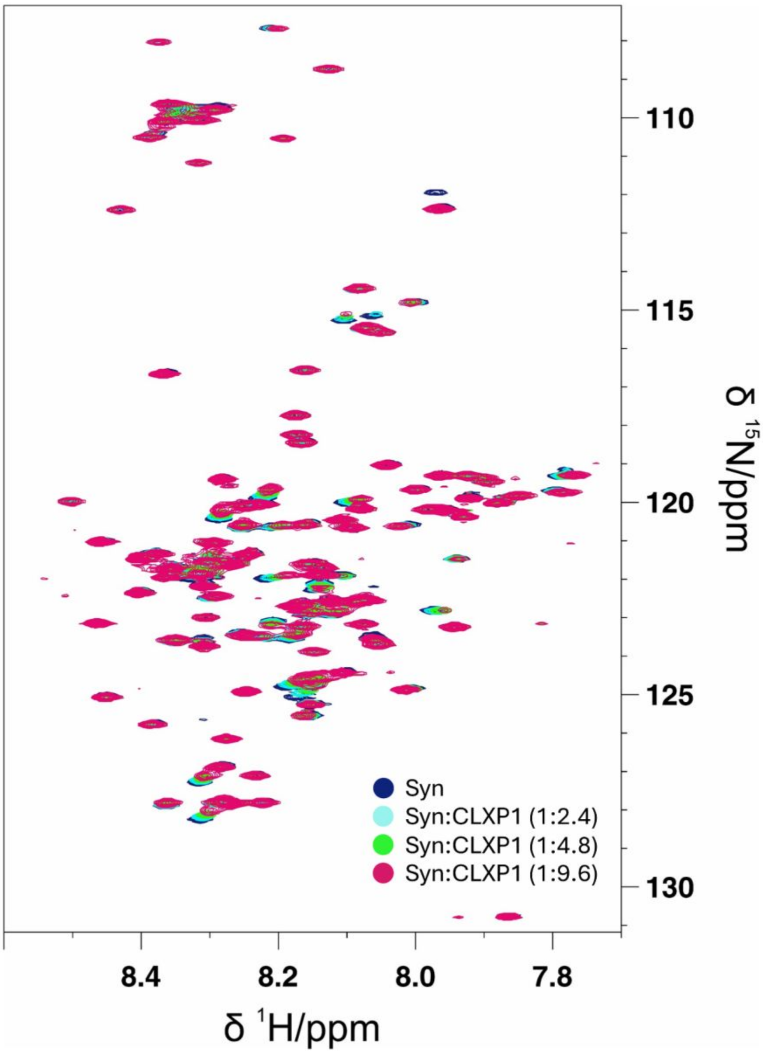

Figure S9. NMR analysis of syn-CLXP1 interaction. Overlay of 2D ^{1}H-^{15}N HSQC spectra of ^{5}N-labelled syn recorded in the absence (dark blue) and in the presence of **CLXP1** at molar ratios of 1:2.4 (light blue), 1:4.8 (green), and 1:9.6 (magenta). NMR spectra were acquired at 298 K on a 900 MHz NMR spectrometer.

A

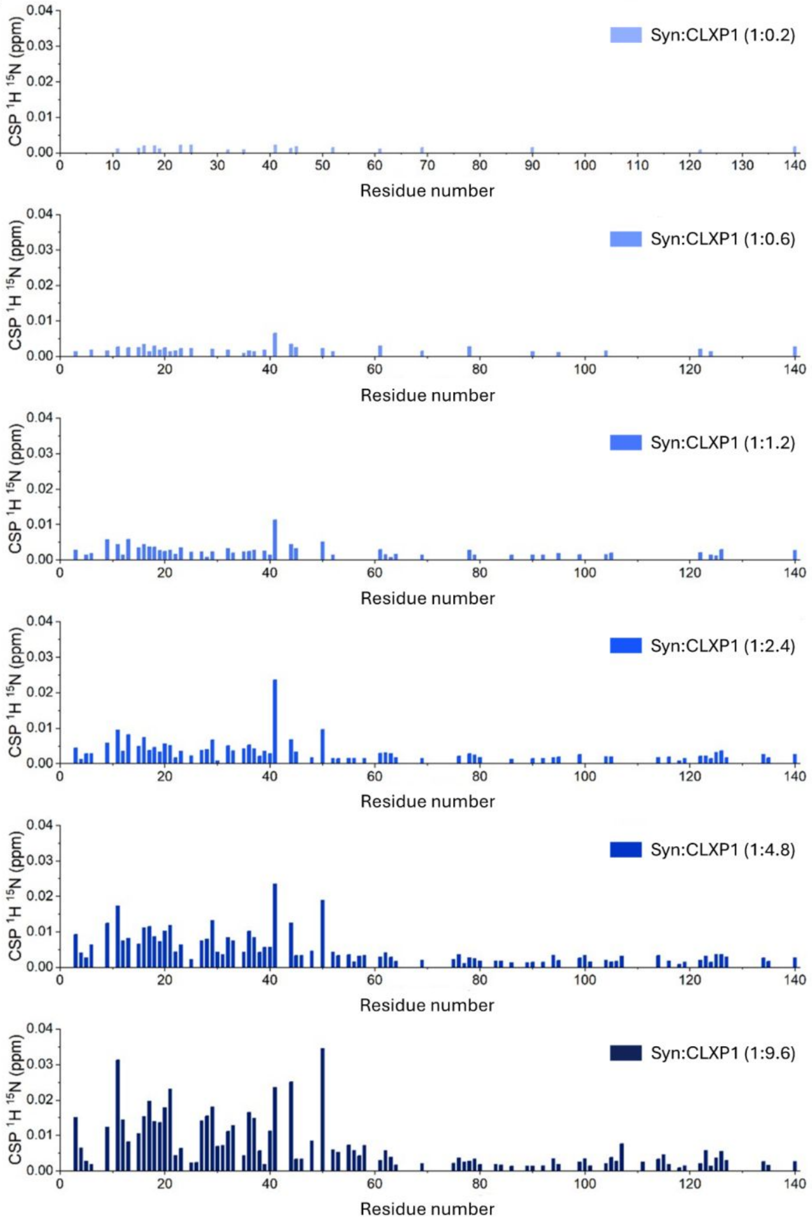

B

Residue	K_d (mM)
S9	0.33 ± 0.18
A11	7.03 ± 0.94
V16	0.77 ± 0.18
A18	1.86 ± 0.99
A29	2.20 ± 0.90
K32	0.90 ± 0.09
T33	8.18 ± 5.74
G36	4.36 ± 1.01
V37	5.64 ± 1.70
T44	11.16 ± 10.15
H50	10.62 ± 2.89

Figure S10. NMR titration analysis of syn-CLXP1 interaction. A) Plot of ^{1}H-^{15}N chemical shift perturbation (CSP) values against the residue number at different titration points (syn:**CLXP1** molar ratios of 1:0.2, 1:0.6, 1:1.2, 1:2,4, 1:4.8, 1:9.6). The CSP value for residue 41, observed at 2.4 equivalents, is also reported for the following titration points, as at higher ligand concentrations the peak intensity decreases, and the signal cannot be reliably detected. **B**) Dissociation constant (K_d) values obtained by fitting the CSP values of selected, well-isolated cross peaks as a function of increasing ligand concentration to a 1:1 interaction model.

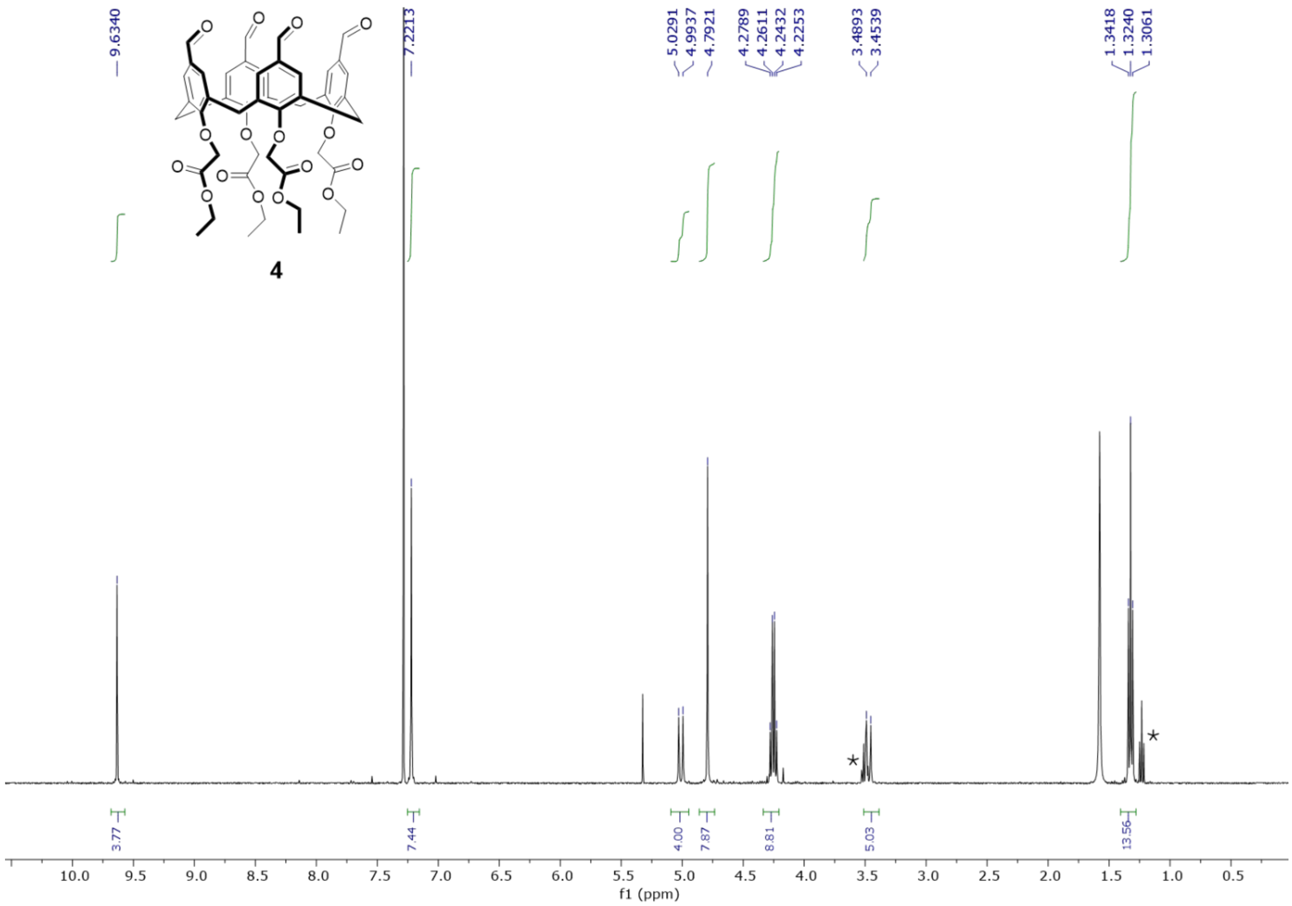

Figure S11. ^{1}H-NMR spectrum of compound **4** (400 MHz, $CDCl_3$, 298 K). *Ethyl ether traces.

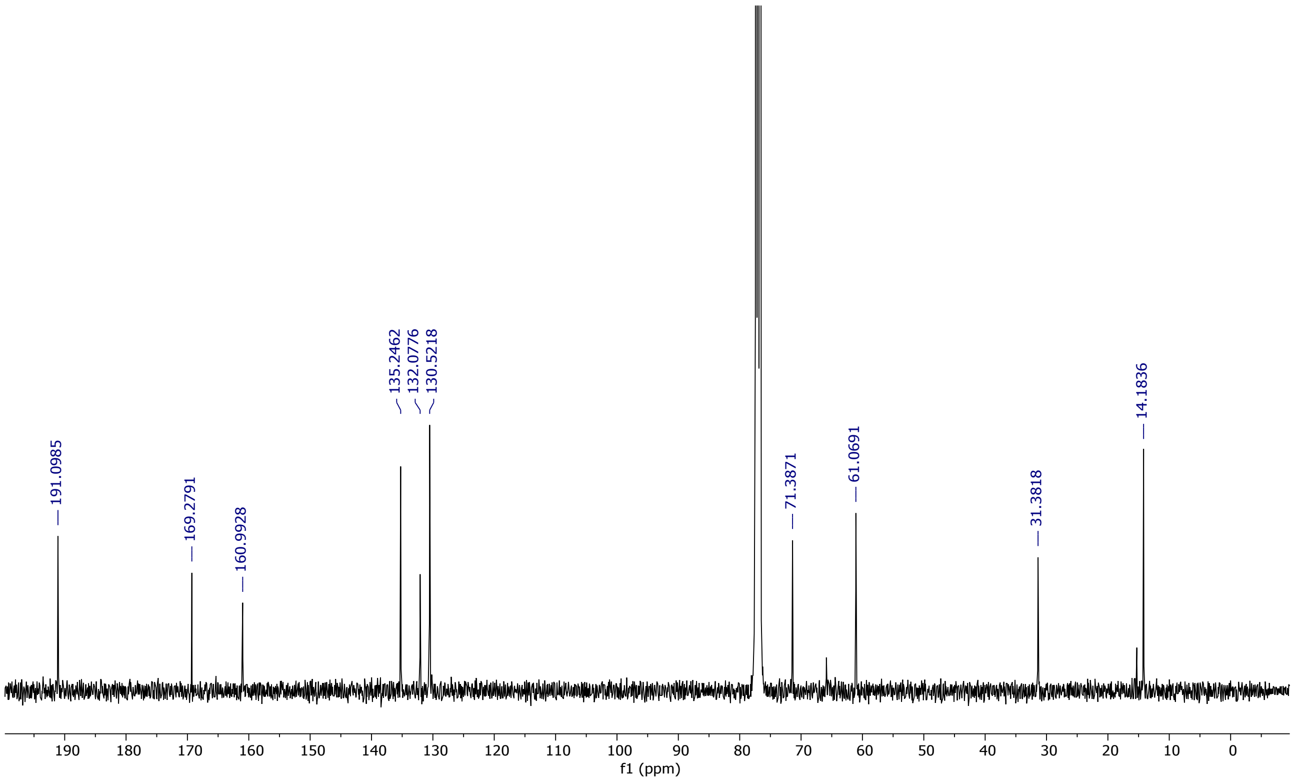

Figure S12. ^{13}C-NMR spectrum of compound **4** (101 MHz, $CDCl_3$, 298 K).

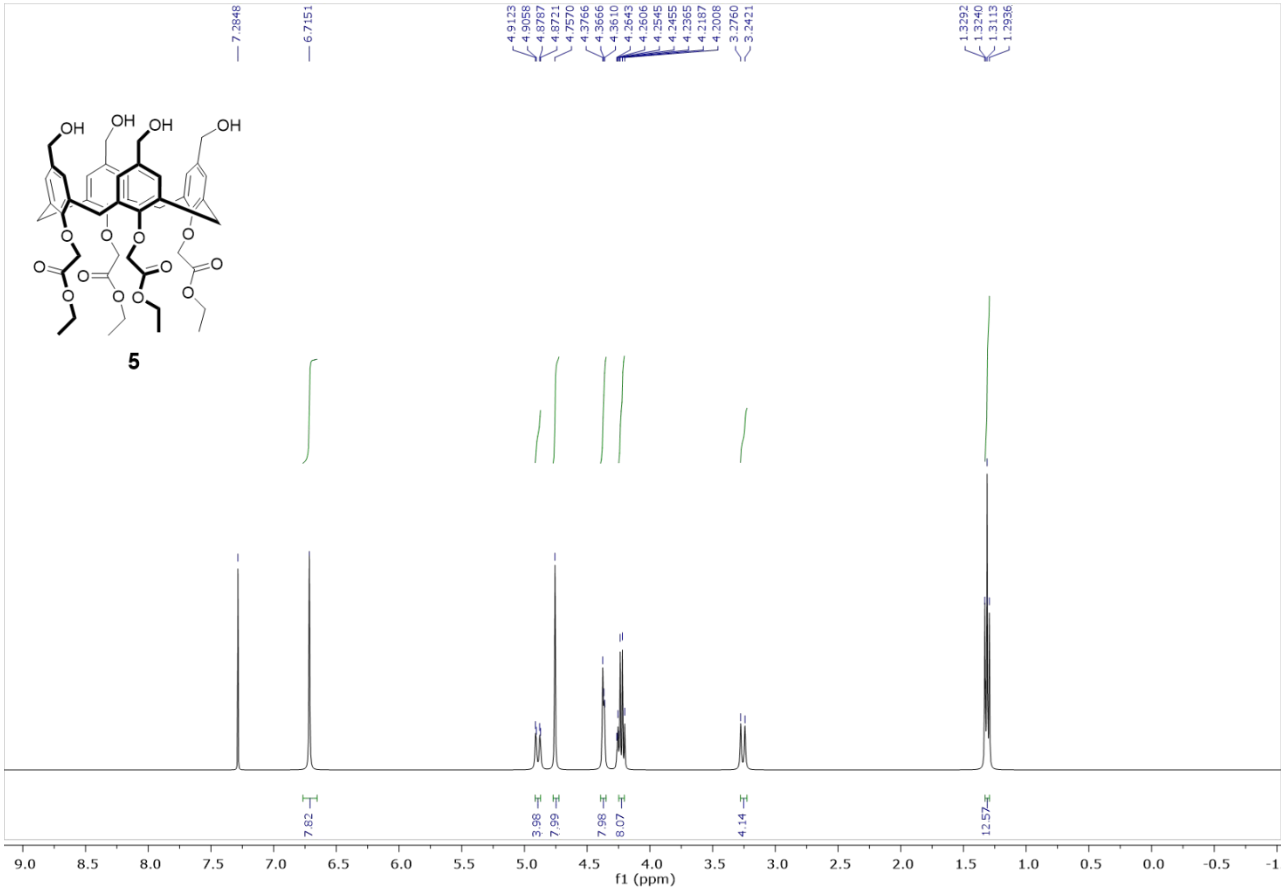

Figure S13. ^{1}H-NMR spectrum of compound **5** (400 MHz, $CDCl_3$, 298 K).

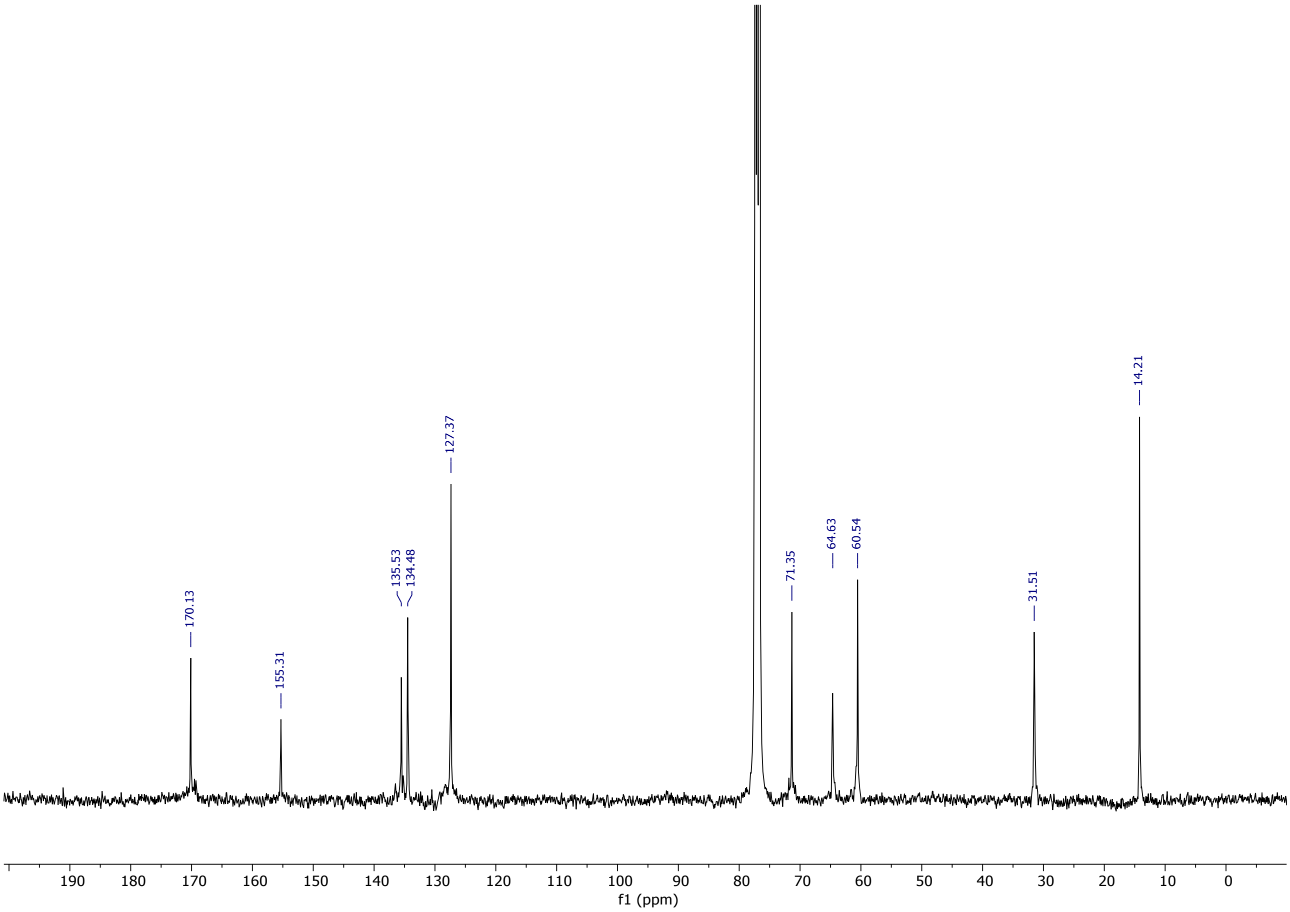

Figure S14. ^{13}C-NMR spectrum of compound **5** (101 MHz, $CDCl_3$, 298 K).

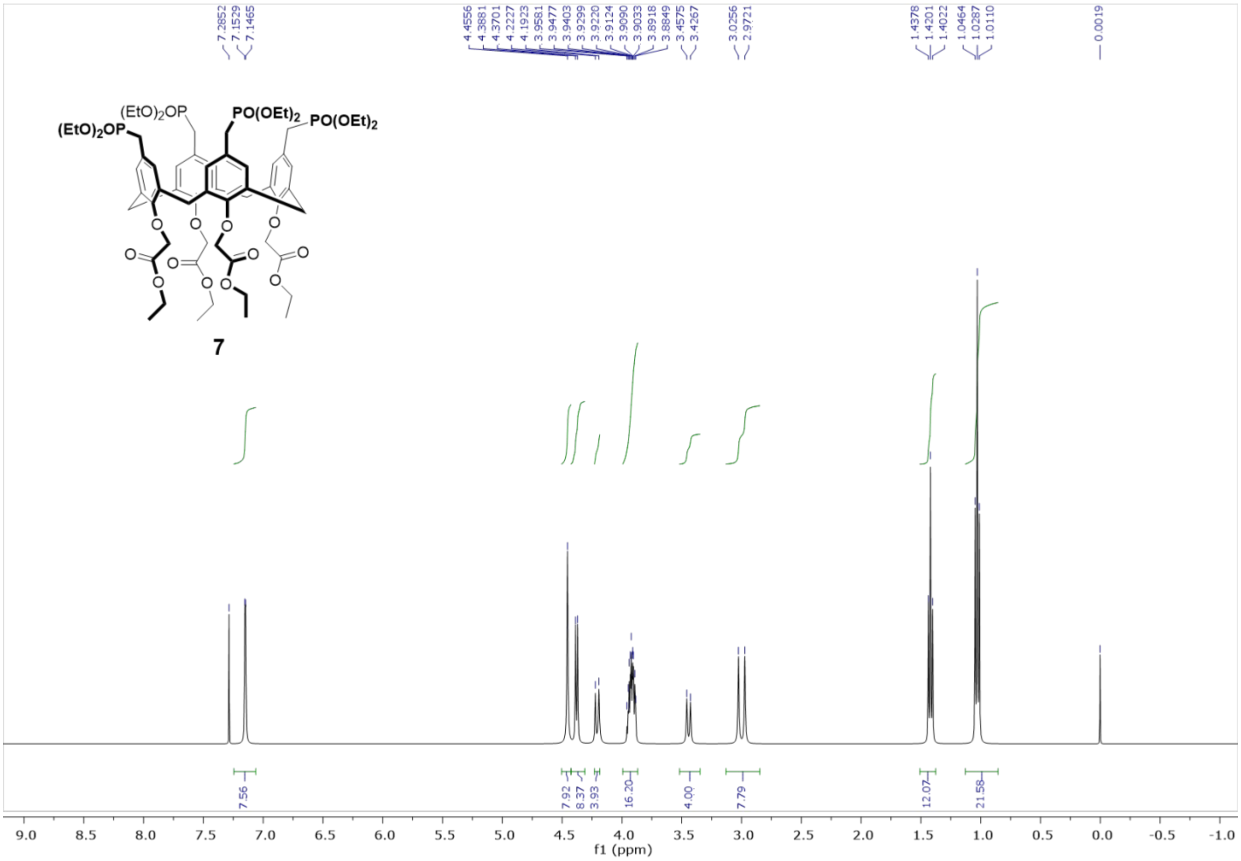

Figure S15. ^{1}H-NMR spectrum of compound **7** (400 MHz, $CDCl_3$, 298 K).

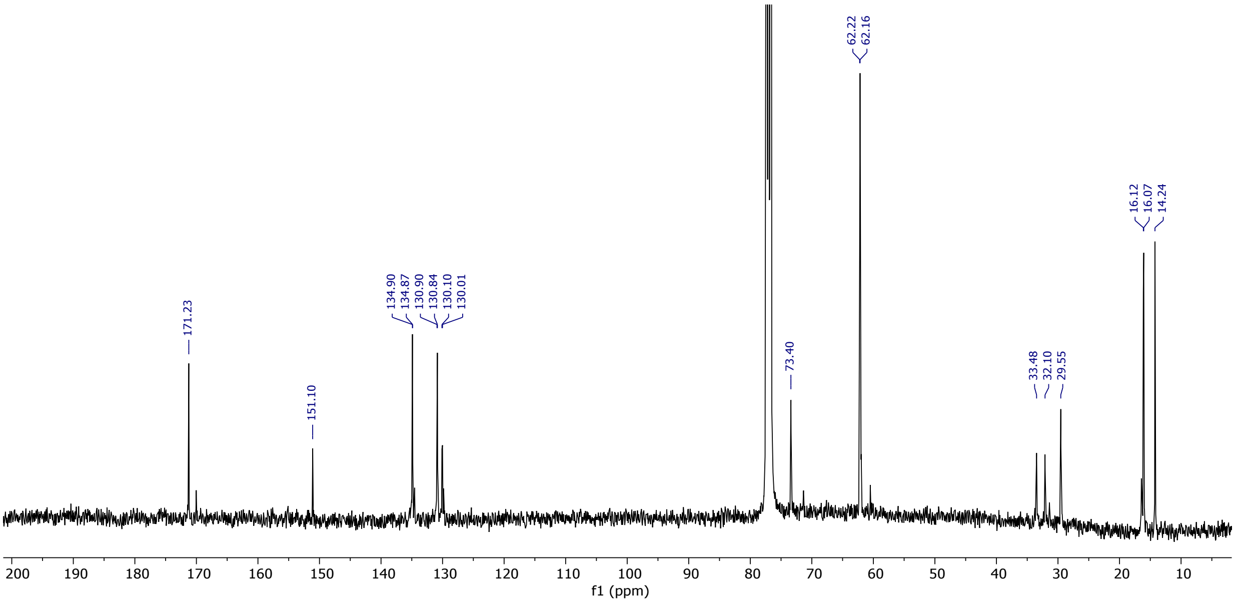

Figure S16. ^{13}C-NMR of compound **7** (101 MHz, $CDCl_3$, 298 K).

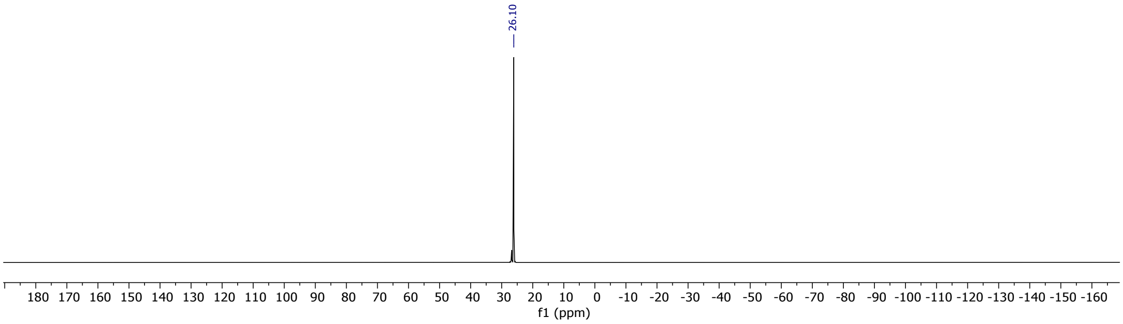

Figure S17. ^{31}P-NMR spectrum of compound **7** (162 MHz, $CDCl_3$, 298 K).

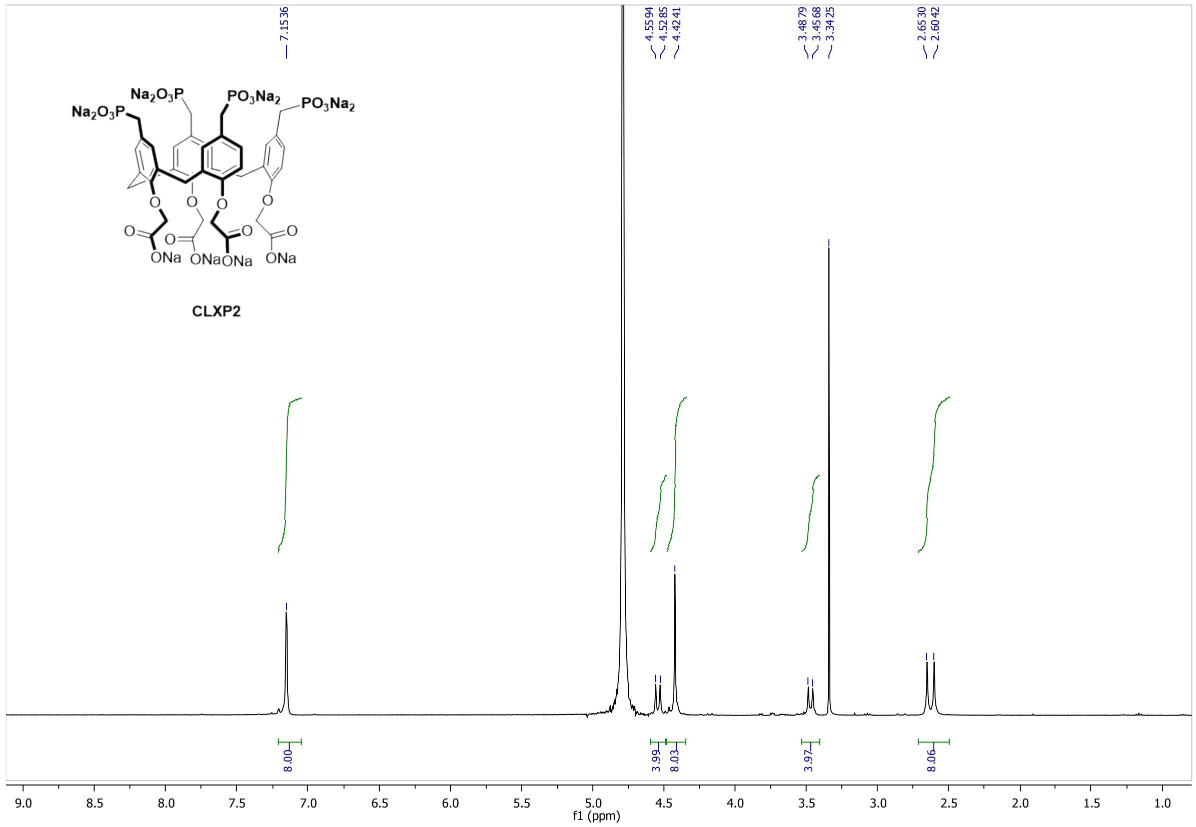

Figure S18. ^{1}H-NMR spectrum of compound **CLXP2** (400 MHz, D_2O, 298 K).

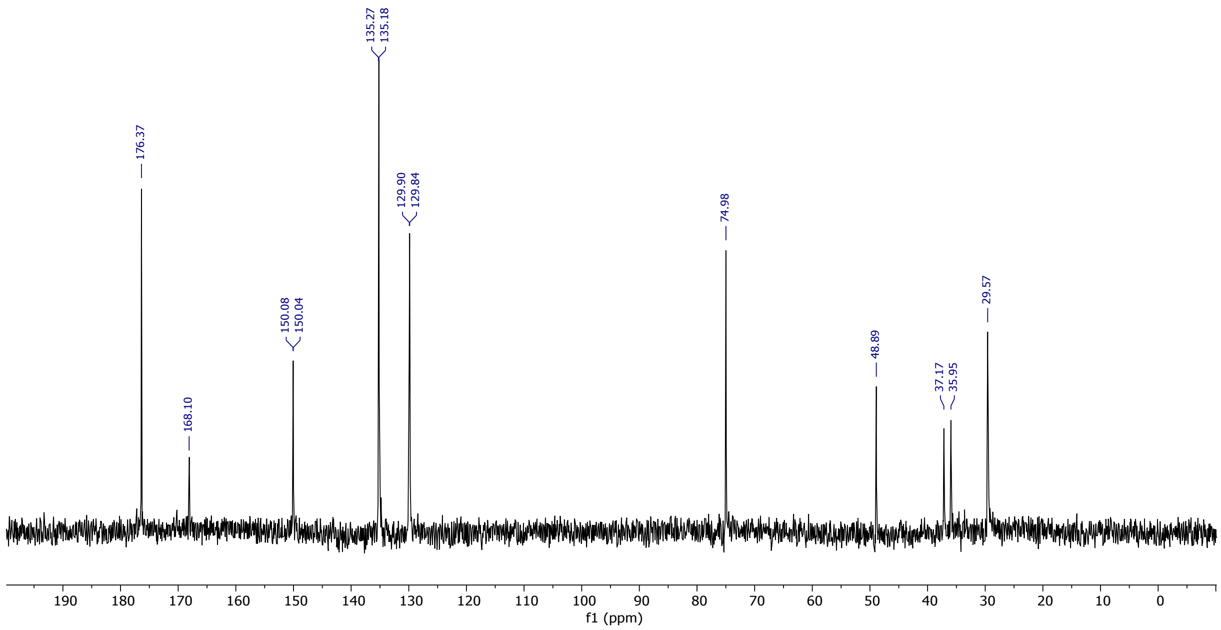

Figure S19. ^{13}C-NMR spectrum of compound **CLXP2** (101 MHz, D_2O, 298 K).

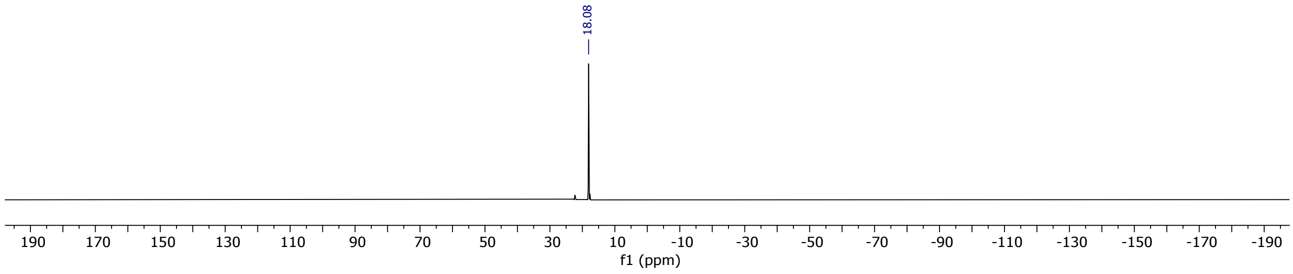

Figure S20. ^{31}P-NMR spectrum of compound **CLXP2** (162 MHz, D_2O, 298 K).

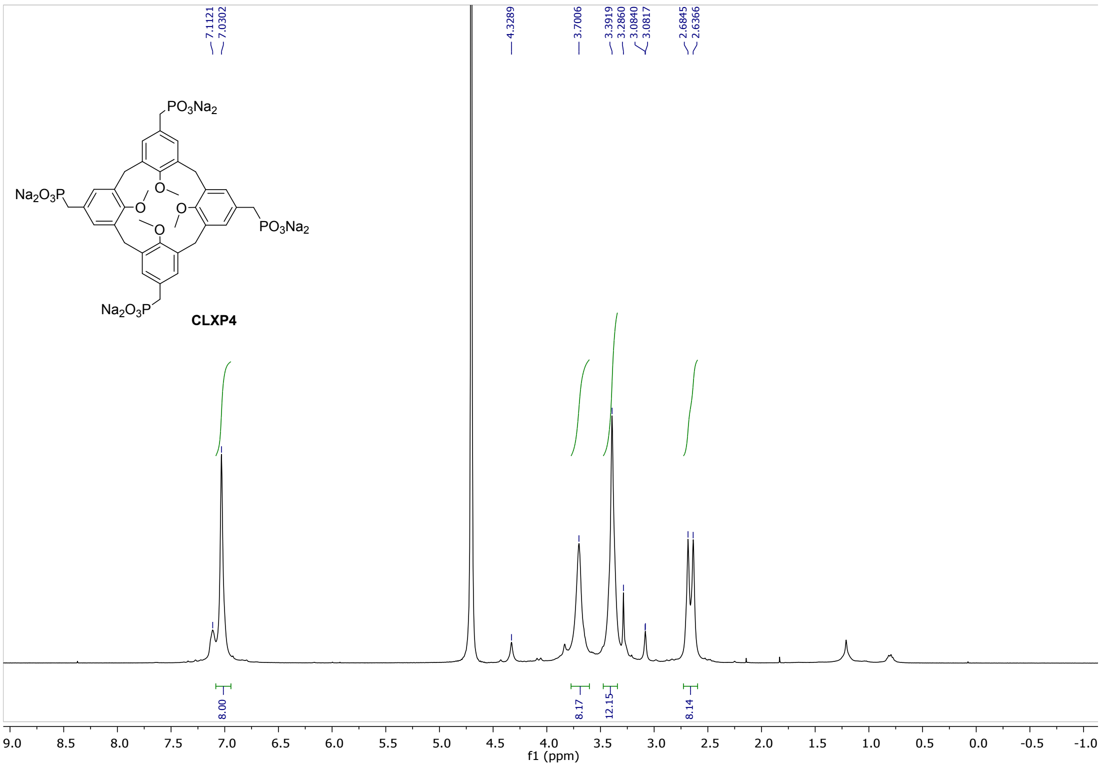

Figure S21. ^{1}H-NMR of compound **CLXP4** (400 MHz, D_2O, 298 K).

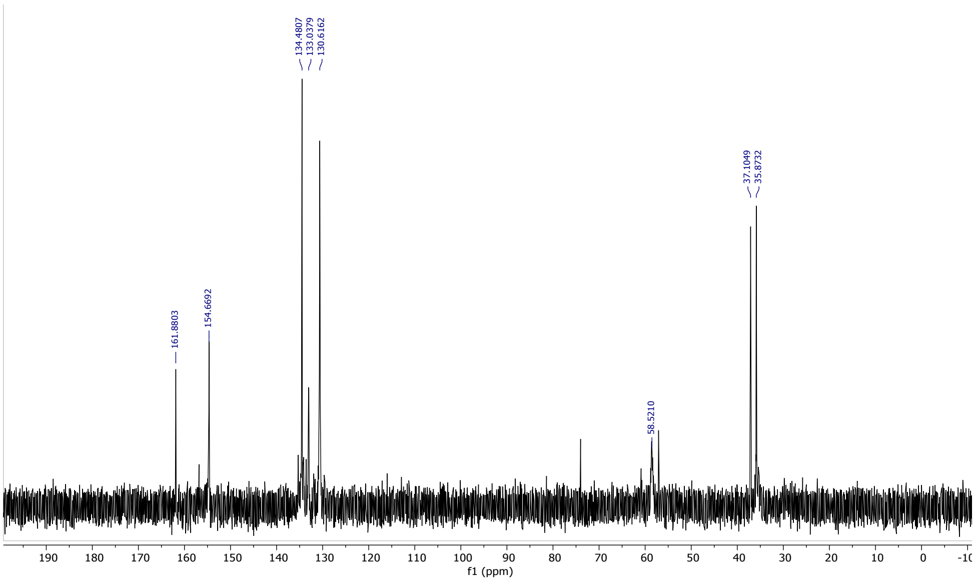

Figure S22. ^{13}C-NMR spectrum of compound **CLXP4** (101 MHz, D_2O, 298 K).

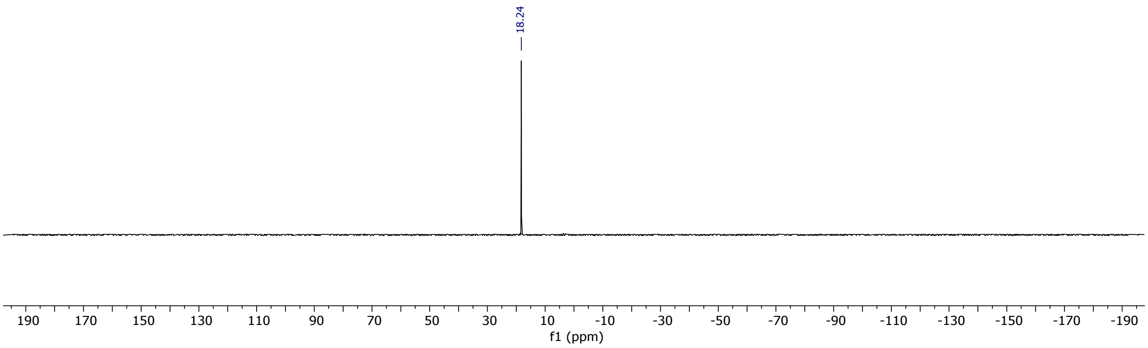

Figure S23. ^{31}P-NMR spectrum of compound **CLXP4** (162 MHz, D_2O, 298 K).

Supporting References

1. Kumar, A.; Sharma, P.; Kalal, B.L.; Chandel, L.K. Synthesis and metal extraction behavior of pyridine and 1,2,4-triazole substituted calix[4]arenes. *J. Incl. Phenom. Macrocycl. Chem.* **2010**, *68*, 369–379.
2. Garai, B.; Shetty, D.; Skorjanc, T.; Gándara, F.; Naleem, N.; Varghese, S.; Kumar, S.; Sharma, S.K.; Baias, M.; Jagannathan, R.; Olson, M.A.; Kirmizialtin, S.; Trabolsi, A. Taming the topology of calix[4]arene-based 2D-covalent organic frameworks: interpenetrated vs noninterpenetrated frameworks and their selective removal of cationic dyes. *J. Am. Chem. Soc.* **2021**, *143* (9), 3407–3415.
3. Arduini, A.; Fabbi, M.; Mantovani, M.; Mirone, L.; Pochini, A.; Secchi, A.; Ungaro, R. Calix[4]arenes blocked in a rigid cone conformation by selective functionalization at the lower rim. *J. Org. Chem.* **1995**, 60 (5), 1454–1457.
4. Nagasaki, T.; Sisido, K.; Arimura, T.; Shinkai, S. Novel conformational isomerism of water-soluble calix[4]arenes. *Tetrahedron* **1992**, *48* (5), 797-804.
5. Mourer, M.; Psychogios, N.; Laumond, G.; Aubertin, A.-M.; Regnouf-de-Vains, J.-B. Synthesis and anti-HIV evaluation of water-soluble calixarene-based bithiazolyl podands. Bioorg. Med. Chem. **2010**, *18* (1), 36-45.
6. Daze, K. D.; Pinter, T.; Beshara, C. S.; Ibraheem, A.; Minaker, S. A.; Ma, M. C. F.; Courtemanche, R. J. M.; Campbell, R. E.; Hof, F. Supramolecular Hosts That Recognize Methyllysines and Disrupt the Interaction between a Modified Histone Tail and Its Epigenetic Reader Protein. *Chem. Sci.* **2012**, *9*, 2695–2699.
7. Gitler, A. D.; Bevis, B. J.; Shorter, J.; Strathearn, K. E.; Hamamichi, S.; Su, L. J.; Caldwell, K. A.; Caldwell, G. A.; Rochet, J. C.; McCaffery, J. M.; Barlowe, C.; Lindquist, S. The Parkinson's disease protein alpha-synuclein disrupts cellular Rab homeostasis. *Proc. Natl. Acad. Sci. U. S. A.* **2008**, *105* (1), 145-150.
8. Ruotolo, R.; De Giorgio, G.; Minato, I.; Bianchi, M. G.; Bussolati, O.; Marmiroli, N. Cerium Oxide Nanoparticles Rescue alpha-Synuclein-Induced Toxicity in a Yeast Model of Parkinson's Disease. *Nanomaterials (Basel)* **2020**, *10* (2).
9. Buttner, S.; Bitto, A.; Ring, J.; Augsten, M.; Zabrocki, P.; Eisenberg, T.; Jungwirth, H.; Hutter, S.; Carmona-Gutierrez, D.; Kroemer, G.; Winderickx, J.; Madeo, F. Functional mitochondria are required for alpha-synuclein toxicity in aging yeast. *J. Biol. Chem.* **2008**, *283* (12), 7554-7560.
10. Tagliaferro, G.; Davighi, M.G., Clemente, F.; Turchi, F.; Schiavina, M.; Matassini, C.; Goti, A.; Morrone, A.; Pierattelli, R.; Cardona, F.; Felli, I.C. Evidence of α-synuclein/glucocerebrosidase dual targeting by iminosugar derivatives. *ACS Chem. Neurosci.* **2025**, *16* (7), 1251–1257.
11. Williamson, M.P. Using chemical shift perturbation to characterise ligand binding. *Prog. Nucl. Magn. Reson. Spectrosc.* **2013**, *73*, 1-16.
